# Synthesis and Bioactivities of Novel 1,3,4-Thiadiazole Derivatives of Glucosides

**DOI:** 10.3389/fchem.2021.645876

**Published:** 2021-03-26

**Authors:** Meihang Chen, Xun Zhang, Daowang Lu, Hairong Luo, Zengyan Zhou, Xufeng Qin, Wenneng Wu, Guoping Zhang

**Affiliations:** ^1^ Colleges of Material and Chemistry Engineering, Tongren University, Tongren, China; ^2^ Colleges of Food and Pharmaceutical Engineering, Guiyang University, Guiyang, China; ^3^ Colleges of Chemistry and Material Science, Huaibei Normal University, Huaibei, China

**Keywords:** thiadiazole, amide, glucoside, synthesis, bioactivity

## Abstract

A series of novel 1,3,4-thiadiazole derivatives of glucosides were synthesized by the starting materials *d*-glucose and 5-amino-1,3,4-thiadiazole-2-thiol in good yields with employing a convergent synthetic route. The results of bioactivities showed that some of the target compounds exhibited good antifungal activities. Especially, compounds **4i** showed higher bioactivities against *Phytophthora infestans* (*P. infestans*), with the EC_50_ values of 3.43, than that of Dimethomorph (5.52 μg/ml). In addition, the target compounds exhibited moderate to poor antibacterial activities against *Xanthomonas oryzae p*v. *oryzae* (*Xoo*)*, Xanthomonas campestris p*v. *citri* (*Xcc*).

## Introduction

Crop disease, caused by fungi, bacteria, viruses, and nematodes and parasitic seed plants, can effect on the biological or non-biological factors of plants causing the phenomenon of a series of morphological, physiological and biochemical pathologic changes, further blocking the normal growth and the development process and the human economic benefits (Zhan et al., 2015). Nowadays, some of the traditional fungicides and bactericides, such as Carbendazim, Kresoxim-methyl, Streptomycin sulfate, Bismerthiazol, etc., have been widely used to prevent and control plant fungal and bacterial diseases. However, long-term using these pesticides could lead to drug resistance, serious ecological, and environmental problem (Aktar et al., 2009). Therefore, development of novel and promising fungicides and bactericides is still an urgent task.

1,3,4-Thiadiazole derivatives have shown extensive biological activities, such as anti-inflammatory (Maddila et al., 2016), anticancer (Yang et al., 2012; Sridhar et al., 2020), antifungal (Alwan et al., 2015; Bhinge et al., 2015; Chudzik et al., 2019), antibacterial (Aggarwal et al., 2012; Taflan et al., 2019; Zhang et al., 2019), and plant growth regulator (Knyazyan et al., 2012) activities. Since 1,3,4-thiadiazole compounds with antibacterial activity was synthesized by Masaki in the 1950s, 1,3,4-thiadiazole pesticides, such as Bismerthiazol and Thiodiazole-copper, have been developed and widely used in agriculture. Recent years, a variety of studies found that amide derivatives containing 1,3,4-thiadiazole thioether moiety showed good antifungal activities against *Fusarium oxysporum* (*F. oxysporum*), *Cytospora mandshurica* (*C. mandshurica*), and *Gibberella zeae* (*G. zeae*) at 50 mg/L (Xie et al., 2016) and exhibited exciting antibacterial activities against *Xanthomonas oryzae p*v. *oryzae* (*Xoo*)*, Xanthomonas campestris p*v. *citri* (*Xcc*)*,* and *Ralstonia solanacearum* (*Rs*) (Chen J. et al., 2019).

Glycosides are secondary metabolites that widely exist in all organs of plants, such as flowers, fruits, leaves, skins, and roots, etc (Gruner et al., 2002), and previous studies found that glycosides had a wide range of pharmacological activities, such as antiviral (Chen W. et al., 2019; Khodair et al., 2019), antibacterial (Mohammed et al., 2019), anticancer (Gurung et al., 2018; Rahim et al., 2020), antioxidant (Jiang et al., 2018; Hawas et al., 2019), and anti-HIV (He et al., 2019) activities. Meanwhile, studies also found that glycoside derivatives showed exceeding inhibitory activities against plant pathogens. For example, Ningnanmycin, an important glycoside biological pesticide, is mainly used in rice seedling blight, soybean root rot, rice stripe disease, apple spot deciduous leaf disease and cucumber powdery mildew (Hu et al., 1997). In addition, it was also found that glycosylation is one of the effective ways to improve the functional activity of active lead compounds and develop new drugs. (Gurung et al., 2018; Wu et al., 2014).

In order to develop new lead compounds with highly bioactivity, in this study, we aimed to introduce a 1,3,4-thiadiazole group into glucosides moiety to design a series of novel 1,3,4-thiadiazole derivatives of glucosides and then evaluate for their antifungal and antibacterial activities. Results indicated that some of the target compounds exhibited good antifungal activities. Especially, the compounds **4i** showed higher bioactivities against *Phytophthora infestans* (*P. infestans*), with the EC_50_ values of 3.43 μg/ml, respectively, than that of Dimethomorph (5.52 μg/ml). In addition, the target compounds showed moderate to poor antibacterial activities against *Xoo* and *Xcc*. As far as we know, this is the first report on the antifungal and antibacterial activities of 1,3,4-thiadiazole derivatives of glucosides.

## Materials and Methods

### Materials and Instruments

Melting points were determined on an XT-4 melting apparatus (Beijing Tech Instrument Co., China). ^1^H NMR and ^13^C NMR spectra were measured on a Bruker AVANCE III TM 400 and HD 600 MHz Digital NMR Spectrometer (Bruker Company, Billerica, MA, US.) in CDCl_3_ as solvent and recorded in relative to internal standard tetramethylsilane. High-resolution mass spectrometry (HRMS) was carried out on an Agilent Technologies 6540 UHD Accurate-Mass Q-TOF LC/MS (Agilent Technologies, Palo Alto, CA, United States). The course of the reactions was monitored by thin-layer chromatography (TLC) analysis on silica gel GF254. All reagents and solvents meet the standards of analytical reagent before use.

### Chemistry

**FIGURE 1:**
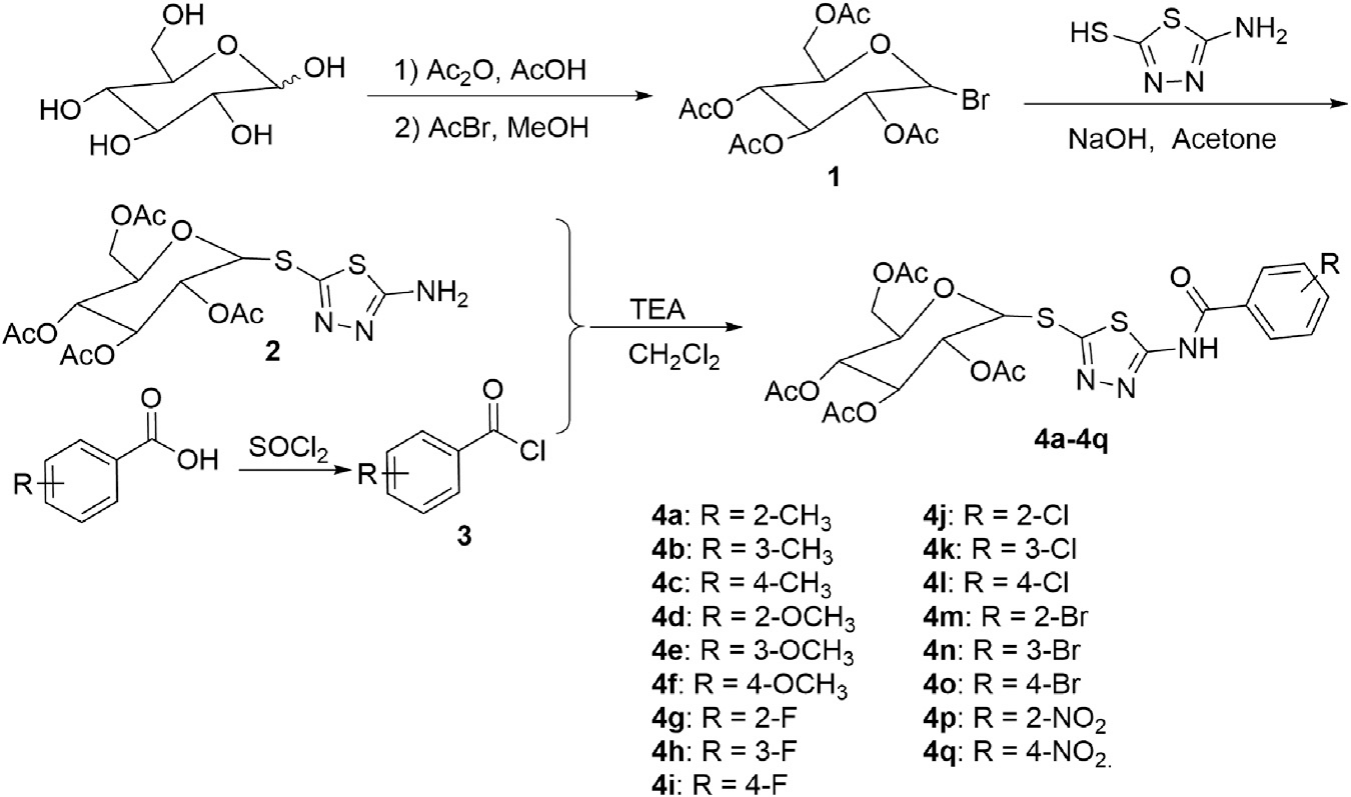
Synthetic route of the target compounds **4a–4q**.

Preparation of 2,3,4,6-tetra-*O*-acetyl-*α*-*d*-glucopyranosyl bromide (**1**)*.* As shown in Figure 1, acetic anhydride (88 ml, 0.9 mol) was added to a solution of *d*-glucose (29.75 g, 0.15 mol) in glacial acetic acid (300 ml) and stirred at room temperature for 20 min. Then, perchloric acid (0.3 ml) was added to the above reaction mixture at room temperature. After TLC analysis showed complete disappearance of *d*-glucose, a solution of acetyl bromide (34 ml, 0.45 mol) in 50 ml CH_2_Cl_2_ was added to the resultant reaction mixture and stirred at room temperature. After the completion of the reaction, the reaction mixture was poured into water and extracted with CH_2_Cl_2_. The organic layer was dried, filtered, and evaporated in vacuo to remove CH_2_Cl_2_. The crude product was recrystallized by a mixture of petroleum ether and diethyl ether (volume ratio 1:2) to afford intermediate **1.** (Scattolin et al., 2020). ^1^H NMR spectral data for intermediate **1** are listed in the Supplementary Material.

Preparation of (2*R*,3*R*,4*S*,5*R*,6*R*)-2-(acetoxymethyl)-6-((5-amino-1,3,4-thiadiazol-2-yl)thio)tetra hydro-2*H*-pyran-3,4,5-triyltriacetate (**2**). A mixture of 2-amino-5-mercapto-1,3,4-thiadiazole (1.33 g, 10.0 mmol), acetone (50 ml), NaOH (0.4 g, 10.0 mmol), and water (10 ml) was stirred for 30 min at room temperature. Then, a solution of intermediate **1** (0.98 g, 10.0 mmol) in 5 ml acetone was added dropwise and continuously stirred at room temperature. After the reaction completed (monitored by TLC), acetone was evaporated in vacuo, the residues were diluted with water, extracted with CH_2_Cl_2_. The combined CH_2_Cl_2_ extract was dried over anhydrous sodium sulfate, evaporated in vacuo and separated by silica gel column chromatography to afford intermediate **2** (Kamat et al., 2007). ^1^H NMR spectral data for intermediate **2** are listed in the Supplementary Material.

General procedure for preparation of the target compounds **4a–4q**. Substituted benzoic acid (1.2 mmol) was added in 2 ml SOCl_2_ and refluxed for about 2 h SOCl_2_ was distilled off in vacuo to obtain intermediates **3**. And then, a solution of intermediate **3** in 2 ml CH_2_Cl_2_ was added dropwise to a mixture of the intermediate **2** (1.0 mmol) and triethylamine (TEA, 1.2 mmol) in 10 ml CH_2_Cl_2_. After the reaction was completed (monitored by TLC), the mixture was diluted with water, the organic layer was dried over anhydrous sodium sulfate, filtered and distilled off in vacuo, and the crude products were recrystallized with isopropanol to afford title compounds **4a–4q**.

(2*R*,3*R*,4*S*,5*R*,6*R*)-2-(acetoxymethyl)-6-((5-(2-methylbenzamido)-1,3,4-thiadiazol-2-yl)thio)tetrahy dro-2*H*-pyran-3,4,5-triyltriacetate (**4a**). White solid; yield 67.1%; m. p. 160–162°C; *R*_f_ = 0.67 (ethyl acetate: petroleum ether, 1:2); IR (KBr, cm^−1^) *ν*: 3,433 (NH), 1747 (COO), 1,678 (CON); ^1^H NMR (400 MHz, CDCl_3_, ppm) *δ*: 7.66 (d, *J* = 8.3 Hz, 1H, Ar-H), 7.49 (d, *J* = 7.6 Hz, 1H, Ar-H), 7.42–7.30 (m, 2H, Ar-H), 5.29 (t, *J* = 10.0 Hz, 1H, H-3´), 5.21–5.06 (m, 3H, H-1´, H-2´, H-4´), 4.34–4.16 (m, 2H, H-5´, H-6´), 3.84–3.80 (m, 1H, H-6´´), 2.55 (s, 3H, CH_3_), 2.15 (s, 3H, CH_3_), 2.10 (s, 3H, CH_3_), 2.04 (s, 3H, CH_3_), 2.02 (s, 3H, CH_3_); ^13^C NMR (150 MHz, CDCl_3_, ppm) *δ*: 170.66 (COCH_3_), 170.17 (COCH_3_), 169.45 (COCH_3_), 169.34 (CONH), 169.28 (thiadiazole-C), 158.58 (thiadiazole-C), 157.90 (Ar-C), 137.27 (Ar-C), 132.05 (Ar-C), 131.56 (Ar-C), 130.75 (Ar-C), 129.18 (Ar-C), 82.52 (C-1´), 75.97 (C-5´), 73.89 (C-3´), 69.28 (C-2´), 67.87 (C-4´), 61.62 (C-6´), 20.74 (CH_3_), 20.61 (CH_3_), 20.59 (CH_3_), 20.02 (CH_3_); HRMS [M + H]^+^ calculated for C_24_H_27_N_3_O_10_S_2_: m/z 582.1230, found 582.1209.

(2*R*,3*R*,4*S*,5*R*,6*R*)-2-(acetoxymethyl)-6-((5-(3-methylbenzamido)-1,3,4-thiadiazol-2-yl)thio)tetra hydro-2*H*-pyran-3,4,5-triyltriacetate (**4b**). White solid; yield 65.3%; m. p. 163–165°C; *R*_f_ = 0.45 (ethyl acetate: petroleum ether, 1:2); IR (KBr, cm^−1^) *ν*: 3,468 (NH), 1749 (COO), 1,666 (CON); ^1^H NMR (400 MHz, CDCl_3_, ppm) *δ*: 7.99 (s, 1H, Ar-H), 7.94 (d, *J* = 7.5 Hz, 1H, Ar-H), 7.51–7.43 (m, 2H, Ar-H), 5.28 (t, *J* = 9.0 Hz, 1H, H-3´), 5.21–4.98 (m, 3H, H-1´, H-2´, H-3´), 4.40–4.05 (m, 2H, H-5´, H-6´), 3.74–3.70 (m, 1H, H-6´´), 2.48 (s, 3H, CH_3_), 2.14 (s, 3H, CH_3_), 2.09 (s, 3H, CH_3_), 2.05 (s, 3H, CH_3_), 2.02 (s, 3H, CH_3_); ^13^C NMR (150 MHz, CDCl_3_, ppm) *δ*: 171.01 (COCH_3_), 170.16 (COCH_3_), 169.41 (COCH_3_), 165.24 (CONH), 162.64 (thiadiazole-C), 155.20 (thiadiazole-C), 139.01 (Ar-C), 134.34 (Ar-C), 130.86 (Ar-C), 128.75 (Ar-C), 125.40 (Ar-C), 84.07 (C-1´), 76.31 (C-5´), 73.58 (C-3´), 69.67 (C-2´), 67.79 (C-4´), 61.77 (C-6´), 21.35 (CH_3_), 20.72 (CH_3_), 20.64 (CH_3_), 20.58 (CH_3_); HRMS [M + H]^+^ calculated for C_24_H_27_N_3_O_10_S_2_: m/z 582.1230, found 582.1208.

(2*R*,3*R*,4*S*,5*R*,6*R*)-2-(acetoxymethyl)-6-((5-(4-methylbenzamido)-1,3,4-thiadiazol-2-yl)thio)tetra hydro-2*H*-pyran-3,4,5-triyltriacetate (**4c**). White solid; yield 75.1%; m. p. 159–161°C; *R*_f_ = 0.63 (ethyl acetate: petroleum ether, 1:2); IR (KBr, cm^−1^) *ν*: 3,433 (NH), 1747 (COO), 1,666 (CON); ^1^H NMR (400 MHz, CDCl_3_, ppm) *δ*: 11.76 (s, 1 H, NH), 8.05 (d, *J* = 8.1 Hz, 2 H, Ar-H), 7.36 (d, *J* = 8.0 Hz, 2H, Ar-H), 5.49 (d, *J* = 3.2 Hz, 1H, H-1´), 5.38 (t, *J* = 10.0 Hz, 1H, H-3´), 5.11 (dd, *J* = 9.9, 3.3 Hz, 1H, H-2´), 5.05 (d, *J* = 10.1 Hz, 1H, H-4´), 4.21 (d, *J* = 6.1 Hz, 2H, H-5´, H-6´´), 4.05–4.01 (m, 1H, H-6´), 2.47 (s, 3H, CH_3_), 2.19 (s, 3H, CH_3_), 2.12 (s, 3H, CH_3_), 2.09 (s, 3H, CH_3_), 2.00 (s, 3H, CH_3_); ^13^C NMR (150 MHz, CDCl_3_, ppm) *δ*: 170.93 (COCH_3_), 170.10 (COCH_3_), 169.35 (COCH_3_), 164.84 (CONH), 162.72 (thiadiazole-C), 155.32 (thiadiazole-C), 144.59 (Ar-C), 129.77 (Ar-C), 128.41 (Ar-C), 84.08 (C-1´), 76.36 (C-5´), 73.60 (C-3´), 69.71 (C-2´), 67.79 (C-4´), 61.76 (C-6´), 21.77 (CH_3_), 20.72 (CH_3_), 20.68 (CH_3_), 20.60 (CH_3_); HRMS [M + H]^+^ calculated for C_24_H_27_N_3_O_10_S_2_: m/z 582.1230, found 582.1209.

(2*R*,3*R*,4*S*,5*R*,6*R*)-2-(acetoxymethyl)-6-((5-(2-methoxybenzamido)-1,3,4-thiadiazol-2yL)thio)tetra hydro-2*H*-pyran-3,4,5-triyltriacetate (**4d**). White solid; yield 68.7%; m. p. 168–170°C; *R*_f_ = 0.33 (ethyl acetate: petroleum ether, 1:2); IR (KBr, cm^−1^) *ν*: 3,433 (NH), 1749 (COO), 1,687 (CON); ^1^H NMR (400 MHz, CDCl_3_, ppm) *δ*: 12.13 (s, 1H, NH), 8.25 (d, *J* = 9.6 Hz, 1H, Ar-H), 7.61 (t, *J* = 8.8 Hz, 1H, Ar-H), 7.19 (t, *J* = 7.6 Hz, 1H, Ar-H), 7.10 (d, *J* = 8.4 Hz, 1H, Ar-H), 5.30 (t, *J* = 9.2 Hz, 1H, H-3´), 5.18–5.08 (m, 3H, H-1´, H-2´, H-4´), 4.32–4.22 (m, 2H, H-5´, H-6´), 4.12 (s, 3H, OCH_3_), 3.86–3.82 (m, 1H, H-6´´), 2.16 (s, 3H, CH_3_), 2.10 (s, 3H, CH_3_), 2.04 (s, 3H, CH_3_), 2.01 (s, 3H, CH_3_); ^13^C NMR (150 MHz, CDCl_3_, ppm) *δ*: 171.01 (COCH_3_), 170.13 (COCH_3_), 169.40 (COCH_3_), 169.38 (COCH_3_), 162.61 (CONH), 161.06 (thiadiazole-C), 158.08 (thiadiazole-C), 155.19 (Ar-C), 135.32 (Ar-C), 132.83 (Ar-C), 121.94 (Ar-C), 118.07 (Ar-C), 111.82 (Ar-C), 84.17 (C-1´), 76.32 (C-5´), 73.63 (C-3´), 69.67 (C-2´), 67.85 (C-4´), 61.83 (C-6´), 56.48 (OCH_3_), 21.77 (CH_3_), 20.72 (CH_3_), 20.68(CH_3_), 20.60 (CH_3_); HRMS [M + H]^+^ calculated for C_24_H_27_N_3_O_11_S_2_: m/z 598.1142, found 598.1161.

(2*R*,3*R*,4*S*,5*R*,6*R*)-2-(acetoxymethyl)-6-((5-(3-methoxybenzamido)-1,3,4-thiadiazol-2-yl)thio)tetra hydro-2*H*-pyran-3,4,5-triyltriacetate (**4e**). White solid; yield 73.2%; m. p. 169–171°C; *R*_f_ = 0.41 (ethyl acetate: petroleum ether, 1:2); IR (KBr, cm^−1^) *ν*: 3,450 (NH), 1749 (COO), 1,666 (CON); ^1^H NMR (400 MHz, CDCl_3_, ppm) *δ*: 11.40 (s, 1H, NH), 7.67 (d, *J* = 7.7 Hz, 1H, Ar-H), 7.58 (s, 1H, Ar-H), 7.48 (t, *J* = 8.0 Hz, 1H, Ar-H), 7.23 (d, *J* = 10.7 Hz, 1H, Ar-H), 5.29 (t, *J* = 9.2 Hz, 1H, H-3´), 5.21–5.04 (m, 3H, H-1´, H-2´, H-4´), 4.34–4.11 (m, 2H, H-5´, H-6´), 3.90 (s, 3H, OCH_3_), 3.75–3.71 (m, 1H, H-6´´), 2.13 (s, 3H, CH_3_), 2.10 (s, 3H, CH_3_), 2.05 (s, 3H, CH_3_), 2.02 (s, 3H, CH_3_); ^13^C NMR (150 MHz, CDCl_3_, ppm) *δ*: 170.54 (COCH_3_), 170.23 (COCH_3_), 169.95 (COCH_3_), 169.50 (CONH), 165.04 (thiadiazole-C), 160.01(thiadiazole-C), 132.19 (Ar-C), 130.08 (Ar-C), 120.61 (Ar-C), 120.05 (Ar-C), 113.18 (Ar-C), 84.44 (C-1´), 75.17 (C-5´), 71.62 (C-3´), 67.16 (C-2´), 66.98 (C-4´), 61.56 (C-6´), 55.60 (OCH_3_), 20.74 (CH_3_), 20.66 (CH_3_), 20.65 (CH_3_), 20.56 (CH_3_); HRMS [M + H]^+^ calculated for C_24_H_27_N_3_O_11_S_2_: m/z 598.1142, found 598.1162.

(2*R*,3*R*,4*S*,5*R*,6*R*)-2-(acetoxymethyl)-6-((5-(4-methoxybenzamido)-1,3,4-thiadiazol-2-yl)thio)tetra hydro-2*H*-pyran-3,4,5-triyltriacetate (**4f**). White solid; yield 75.0%; m. p. 166–168°C; *R*_f_ = 0.52 (ethyl acetate: petroleum ether, 1:2); IR (KBr, cm^−1^) *ν*: 3,462 (NH), 1747 (COO), 1,664 (CON); ^1^H NMR (400 MHz, CDCl_3_, ppm) *δ*: 8.04 (d, *J* = 8.9 Hz, 2H, Ar-H), 7.04 (d, *J* = 8.9 Hz, 2H, Ar-H), 5.28 (t, *J* = 9.2 Hz, 1H, H-3´), 5.20–5.04 (m, 3H, H-1´, H-2´, H-4´), 4.32–4.17 (m, 2H, H-5´, H-6´), 3.92 (s, 3H, OCH_3_), 3.83–3.77 (m, 1H, H-6´´), 2.15 (s, 3H, CH_3_), 2.10 (s, 3H, CH_3_), 2.04 (s, 3H, CH_3_), 2.01 (s, 3H, CH_3_); ^13^C NMR (150 MHz, CDCl_3_, ppm) *δ*: 170.58 (COCH_3_), 170.44 (COCH_3_), 169.87(CONH), 169.79(thiadiazole-C), 163.63 (thiadiazole-C), 155.45 (Ar-C), 131.02 (Ar-C), 123.57 (Ar-C), 114.53 (Ar-C), 83.14 (C-1´), 74.69 (C-5´), 71.15 (C-3´), 68.07 (C-2´), 67.31 (C-4´), 62.42 (C-6´), 56.07 (OCH_3_), 20.91 (CH_3_), 20.88 (CH_3_), 20.79 (CH_3_), 20.76 (CH_3_); HRMS [M + H]^+^ calculated for C_24_H_27_N_3_O_11_S_2_: m/z 598.1142, found 598.1162.

(2*R*,3*R*,4*S*,5*R*,6*R*)-2-(acetoxymethyl)-6-((5-(2-fluorobenzamido)-1,3,4-thiadiazol-2-yl)thio)tetrahy dro-2*H*-pyran-3,4,5-triyltriacetate (**4g**). White solid; yield 55.4%; m. p. 173–175°C; *R*_f_ = 0.61 (ethyl acetate: petroleum ether, 1:2); IR (KBr, cm^−1^) *ν*: 3,421 (NH), 1749 (COO), 1,676 (CON); ^1^H NMR (400 MHz, CDCl_3_, ppm) *δ*: 10.29 (s, 1H, NH), 8.17 (t, *J* = 7.8 Hz, 1H, Ar-H), 7.66 (d, *J* = 7.4 Hz, 1H, Ar-H), 7.38 (t, *J* = 8.0 Hz, 1H, Ar-H), 7.31–7.23 (m, 1H, Ar-H), 5.35–5.23 (m, 1H, H-3´), 5.19–5.08 (m, 3H, H-1´, H-2´, H-4´), 4.31 (dd, *J* = 12.5, 5.0 Hz, 1H, H-5´), 4.21 (dd, *J* = 12.5, 2.0 Hz, 1H, H-6´), 3.85–3.83 (m, 1H, H-6´´), 2.16 (s, 3H, CH_3_), 2.10 (s, 3H, CH_3_), 2.04 (s, 3H, CH_3_), 2.01 (s, 3H, CH_3_); ^13^C NMR (150 MHz, CDCl_3_, ppm) *δ*: 170.93 (COCH_3_), 170.07 (COCH_3_), 169.34 (COCH_3_), 169.29 (COCH_3_), 164.45 (CONH), 163.34 (thiadiazole-C), 155.18 (thiadiazole-C), 132.36 (Ar-C), 130.21 (Ar-C), 129.51 (Ar-C), 128.78 (Ar-C), 83.81 (C-1´), 76.48 (C-5´), 73.57 (C-3´), 69.68 (C-2´), 67.69 (C-4´), 61.69 (C-6´), 20.73 (CH_3_), 20.60 (CH_3_); HRMS [M + H]^+^ calculated for C_23_H_24_FN_3_O_10_S_2_: m/z 586.0932, found 586.0964.

(2R,3R,4S,5R, 6R)-2-(acetoxymethyl)-6-((5-(3-fluorobenzamido)-1,3,4-thiadiazol-2-yl)thio)tetra hydro-2*H*-pyran-3,4,5-triyltriacetate (**4h**). White solid; yield 70.2%; m. p. 174–176°C; *R*_f_ = 0.63 (ethyl acetate: petroleum ether, 1:2); IR (KBr, cm^−1^) *ν*: 3,435 (NH), 1749 (COO), 1,670 (CON); ^1^H NMR (400 MHz, CDCl_3_, ppm) *δ*: 12.30 (s, 1H, NH), 8.29 (s, 1H, Ar-H), 17–8.02 (M, 1H, Ar-H), 7.81 (d, *J* = 8.7 Hz, 1H, Ar-H), 7.46 (t, *J* = 7.9 Hz, 1H, Ar-H), 5.32–5.28 (m, 1H, H-3´), 5.20–5.05 (m, 3H, H-1´, H-2´, H-4´), 4.28 (dd, *J* = 12.5, 5.0 Hz, 1H, H-5´), 4.24–4.11 (m, 1H, H-6´), 3.78–3.73 (m, 1H, H-6´´), 2.14 (s, 3H, CH_3_), 2.09 (s, 3H, CH_3_), 2.05 (s, 3H, CH_3_), 2.02 (s, 3H, CH_3_); ^13^C NMR (150 MHz, CDCl_3_, ppm) *δ*: 170.93 (COCH_3_), 170.07 (COCH_3_), 169.34 (COCH_3_), 169.29 (COCH_3_), 164.45 (CONH), 163.34 (thiadiazole-C), 155.18 (thiadiazole-C), 132.36 (Ar-C), 130.21 (Ar-C), 129.51 (Ar-C), 128.78 (Ar-C), 83.81(C-1´), 76.48 (C-5´), 73.57 (C-3´), 69.68 (C-2´), 67.69 (C-4´), 61.69 (C-6´), 20.73 (CH_3_), 20.60 (CH_3_); HRMS [M + H]^+^ calculated for C_23_H_24_FN_3_O_10_S_2_: m/z 586.0932, found 586.0963.

(2*R*,3*R*,4*S*,5*R*,6*R*)-2-(acetoxymethyl)-6-((5-(4-fluorobenzamido)-1,3,4-thiadiazol-2-yl)thio)tetra hydro-2*H*-pyran-3,4,5-triyltriacetate (**4i**). White solid; yield 65.8%; m. p. 170–172°C; *R*_f_ = 0.64 (ethyl acetate: petroleum ether, 1:2); IR (KBr, cm^−1^) *ν*: 3,475 (NH), 1751 (COO), 1,676 (CON); ^1^H NMR (400 MHz, CDCl_3_, ppm) *δ*: 12.54 (s, 1H, NH), 8.36 (d, *J* = 8.2 Hz, 2H, Ar-H), 7.85 (d, *J* = 8.3 Hz, 2H, Ar-H), 5.28 (t, *J* = 9.3 Hz, 1H, H-3´), 5.18–5.09 (m, 2H H-1´, H-2´), 5.00 (d, *J* = 10.0 Hz, 1H, H-4´), 4.33 (dd, *J* = 12.5, 4.8 Hz, 1H, H-5´), 4.23 (dd, *J* = 12.5, 2.0 Hz, 1H, H-6´), 3.86–3.82 (m, 1H, H-6´´), 2.17 (s, 3H, CH_3_), 2.09 (s, 3H, CH_3_), 2.04 (s, 3H, CH_3_), 2.01 (s, 3H, CH_3_); ^13^C NMR (150 MHz, CDCl_3_, ppm) *δ*: 170.31 (COCH_3_), 170.21 (COCH_3_), 170.00 (COCH_3_), 169.58 (COCH_3_), 166.22 (CONH), 159.08 (thiadiazole-C), 158.51 (thiadiazole-C), 134.35 (Ar-C), 134.29 (Ar-C), 115.52 (Ar-C), 115.38 (Ar-C), 83.14 (C-1´), 74.72(C-5´), 71.83(C-3´), 67.12(C-2´), 66.83(C-4´), 61.34 (C-6´), 20.73 (CH_3_), 20.67 (CH_3_), 20.65 (CH_3_), 20.59 (CH_3_); HRMS [M + H]^+^ calculated for C_23_H_24_FN_3_O_10_S_2_: m/z 586.0932, found 586.0962.

(2*R*,3*R*,4*S*,5*R*,6*R*)-2-(acetoxymethyl)-6-((5-(2-chlorobenzamido)-1,3,4-thiadiazol-2-yl)thio)tetra hydro-2*H*-pyran-3,4,5-triyltriacetate (**4j**). White solid; yield 70.1%; m. p. 178–180°C; *R*_f_ = 0.55 (ethyl acetate: petroleum ether, 1:2); IR (KBr, cm^−1^) *ν*: 3,441 (NH), 1747 (COO), 1,668 (CON); ^1^H NMR (400 MHz, CDCl_3_, ppm) *δ*: 10.48 (s, 1H, NH), 7.90 (d, *J* = 8.4 Hz, 1H, Ar-H), 7.57–7.41 (m, 3H, Ar-H), 5.31–5.11 (m, 4H, H-3´, H-1´, H-2´, H-4´), 4.32–4.19 (m, 2H, H-5´, H-6´), 3.86–3.81 (m, 1H, H-6´´), 2.15 (s, 3H, CH_3_), 2.10 (s, 3H, CH_3_), 2.04 (s, 3H, CH_3_), 2.01 (s, 3H, CH_3_); ^13^C NMR (150 MHz, CDCl_3_, ppm) *δ*: 170.93 (COCH_3_), 170.12 (COCH_3_), 169.37 (COCH_3_), 169.33 (COCH_3_), 163.64 (CONH), 160.98 (thiadiazole-C), 155.66 (thiadiazole-C), 133.34 (Ar-C), 131.70 (Ar-C), 131.24 (Ar-C), 131.19 (Ar-C), 131.02 (Ar-C), 127.60 (Ar-C), 84.00 (C-1´), 76.39 (C-5´), 73.57 (C-3´), 69.65 (C-2´), 67.80 (C-4´), 61.75 (C-6´), 20.78 (CH_3_), 20.68 (CH_3_), 20.60 (CH_3_); HRMS [M + H]^+^ calculated for C_23_H_24_ClN_3_O_10_S_2_: m/z 602.0641, found 602.0663.

(2*R*,3*R*,4*S*,5*R*,6*R*)-2-(acetoxymethyl)-6-((5-(3-chlorobenzamido)-1,3,4-thiadiazol-2-yl)thio)tetra hydro-2*H*-pyran-3,4,5-triyltriacetate (**4k**). White solid; yield 65.3%; m. p. 179–180°C; *R*_f_ = 0.66 (ethyl acetate: petroleum ether, 1:2); IR (KBr, cm^−1^) *ν*: 3,442 (NH), 1749 (COO), 1,674 (CON); ^1^H NMR (400 MHz, CDCl_3_, ppm) *δ*: 12.34 (s, 1H, NH), 8.16 (s, 1H, Ar-H), 8.08 (d, *J* = 7.8 Hz, 1H, Ar-H), 7.66 (d, *J* = 8.0 Hz, 1H, Ar-H), 7.52 (t, *J* = 7.9 Hz, 1H, Ar-H), 5.29 (t, *J* = 9.2 Hz, 1H, H-3´), 5.22–5.06 (m, 3H, H-1´, H-2´, H-4´), 4.37–4.10 (m, 2H, H-5´, H-6´), 3.84–3.81 (m, 1H, H-6´´), 2.14 (s, 3H, CH_3_), 2.09 (s, 3H, CH_3_), 2.05 (s, 3H, CH_3_), 2.02 (s, 3H, CH_3_); ^13^C NMR (150 MHz, CDCl_3_, ppm) *δ*: 170.89 (COCH_3_), 170.12 (COCH_3_), 169.35 (COCH_3_), 169.33 (COCH_3_), 164.26 (CONH), 163.15 (thiadiazole-C), 155.75 (thiadiazole-C), 135.22 (Ar-C), 133.60 (Ar-C), 132.57 (Ar-C), 130.32 (Ar-C), 128.74 (Ar-C), 126.94 (Ar-C), 83.98 (C-1´), 76.37 (C-5´), 73.52 (C-3´), 69.64 (C-2´), 67.79 (C-4´), 61.75 (C-6´), 20.74 (CH_3_), 20.66 (CH_3_), 20.60 (CH_3_); HRMS [M + H]^+^ calculated for C_23_H_24_ClN_3_O_10_S_2_: m/z 602.0641, found 602.0661.

(2*R*,3*R*,4*S*,5*R*,6*R*)-2-(acetoxymethyl)-6-((5-(4-chlorobenzamido)-1,3,4-thiadiazol-2-yl)thio)tetra hydro-2*H*-pyran-3,4,5-triyltriacetate (**4l**). White solid; yield 78.5%; m. p. 178–180°C; *R*_f_ = 0.48 (ethyl acetate: petroleum ether, 1:2); IR (KBr, cm^−1^) *ν*: 3,450 (NH), 1751 (COO), 1,672 (CON); ^1^H NMR (400 MHz, CDCl_3_, ppm) *δ*: 11.88 (s, 1H, NH), 8.13 (d, *J* = 8.6 Hz, 2H, Ar-H), 7.55 (d, *J* = 8.6 Hz, 2H, Ar-H), 5.29 (t, *J* = 9.2 Hz, 1H, H-3´), 5.21–5.00 (m, 3H, H-1´, H-2´, H-4´), 4.36–4.25 (m, 1H, H-5´), 4.20 (dd, *J* = 12.5, 2.0 Hz, 1H, H-6´), 3.84–3.81 (m, 1H, H-6´´), 2.15 (s, 3H, CH_3_), 2.11 (s, 3H, CH_3_), 2.04 (s, 3H, CH_3_), 2.01 (s, 3H, CH_3_); ^13^C NMR (150 MHz, CDCl_3_, ppm) *δ*: 170.59 (COCH_3_), 170.26 (COCH_3_), 169.92 (COCH_3_), 169.48 (COCH_3_), 164.45 (CONH), 163.56 (thiadiazole-C), 155.49 (thiadiazole-C), 139.98 (Ar-C), 130.24 (Ar-C), 129.32 (Ar-C), 129.06 (Ar-C), 84.26 (C-1´), 75.32 (C-5´), 71.62 (C-3´), 67.09 (C-2´), 66.90 (C-4´), 61.66 (C-6´), 20.79 (CH_3_), 20.70 (CH_3_), 20.68 (CH_3_), 20.58 (CH_3_); HRMS [M + H]^+^ calculated for C_23_H_24_ClN_3_O_10_S_2_: m/z 602.0641, found 602.0664.

(2*R*,3*R*,4*S*,5*R*,6*R*)-2-(acetoxymethyl)-6-((5-(2-bromobenzamido)-1,3,4-thiadiazol-2-yl)thio)tetra hydro-2*H*-pyran-3,4,5-triyltriacetate (**4m**). White solid; yield 69.4%; m. p. 190–192°C; *R*_f_ = 0.65 (ethyl acetate: petroleum ether, 1:2); IR (KBr, cm^−1^) *ν*: 3,473 (NH), 1745 (COO), 1,689 (CON); ^1^H NMR (400 MHz, CDCl_3_, ppm) *δ*: 11.35 (s, 1H, NH), 7.76 (d, *J* = 7.2 Hz, 1H, Ar-H), 7.72 (d, *J* = 7.4 Hz, 1H, Ar-H), 7.51–7.44 (m, 2H, Ar-H), 5.29 (t, *J* = 9.2, 1H, H-3´), 5.16–5.08 (m, 3H, H-1´, H-2´, H-4´), 4.29 (dd, *J* = 12.5, 5.0 Hz, 1H, H-5´), 4.18 (dd, *J* = 12.5, 2.0 Hz, 1H, H-6´), 3.82–3.78 (m, 1H, H-6´´), 2.14 (s, 3H, CH_3_), 2.10 (s, 3H, CH_3_), 2.05 (s, 3H, CH_3_), 2.02 (s, 3H, CH_3_); ^13^C NMR (150 MHz, CDCl_3_, ppm) *δ*: 170.62 (COCH_3_), 170.25 (COCH_3_), 169.99 (COCH_3_), 169.49 (COCH_3_), 164.27 (CONH), 163.20 (thiadiazole-C), 155.98 (thiadiazole-C), 135.18 (Ar-C), 133.49 (Ar-C), 132.58 (Ar-C), 130.28 (Ar-C), 128.69 (Ar-C), 126.99 (Ar-C), 84.45 (C-1´), 75.22 (C-5´), 71.60 (C-3´), 67.17 (C-2´), 66.96 (C-4´), 61.64 (C-6´), 20.73 (CH_3_), 20.67 (CH_3_), 20.57 (CH_3_); HRMS [M + H]^+^ calculated for C_23_H_24_BrN_3_O_10_S_2_: m/z 646.0171, found 646.0161.

(2*R*,3*R*,4*S*,5*R*,6*R*)-2-(acetoxymethyl)-6-((5-(3-bromobenzamido)-1,3,4-thiadiazol-2-yl)thio)tetrahy dro-2*H*-pyran-3,4,5-triyltriacetate (**4n**). White solid; yield 60.2%; m. p. 191–193°C; *R*_f_ = 0.70 (ethyl acetate: petroleum ether, 1:2); IR (KBr, cm^−1^) *ν*: 3,475 (NH), 1753 (COO), 1,676 (CON); ^1^H NMR (400 MHz, CDCl_3_, ppm) *δ*: 12.30 (s, 1H, NH), 8.29 (s, 1H, Ar-H), 8.11 (d, *J* = 9.2 Hz, Ar-H), 7.81 (d, *J* = 8.7 Hz, 1H, Ar-H), 7.46 (t, *J* = 7.9 Hz, 1H, Ar-H), 5.29 (t, *J* = 9.3 Hz, 1H, H-3´), 5.17–5.12 (m, 3H, H-1´, H-2´, H-4´), 4.28 (dd, *J* = 12.5, 5.0 Hz, 1H, H-5´), 4.19–4.16 (m, 1H, H-6´), 3.78–3.73 (m, 1H, H-6´´), 2.14 (s, 3H, CH_3_), 2.09 (s, 3H, CH_3_), 2.05 (s, 3H, CH_3_), 2.02 (s, 3H, CH_3_); ^13^C NMR (150 MHz, CDCl_3_, ppm) *δ*: 170.87 (COCH_3_), 170.11 (COCH_3_), 169.34 (COCH_3_), 169.32 (COCH_3_), 164.09 (CONH), 163.06 (thiadiazole-C), 155.82 (thiadiazole-C), 136.53 (Ar-C), 132.78 (Ar-C), 131.53 (Ar-C), 130.56 (Ar-C)**,** 127.36 (Ar-C)**,** 123.19 (Ar-C), 84.00 (C-1´), 76.39 (C-5´), 73.52 (C-3´), 69.67 (C-2´), 67.80 (C-4´), 61.76 (C-6´), 20.74 (CH_3_), 20.68 (CH_3_), 20.60(CH_3_); HRMS [M + H]^+^ calculated for C_23_H_24_BrN_3_O_10_S_2_: m/z 646.0171, found 646.0162.

(2*R*,3*R*,4*S*,5*R*,6*R*)-2-(acetoxymethyl)-6-((5-(4-bromobenzamido)-1,3,4-thiadiazol-2-yl)thio)tetrahy dro-2*H*-pyran-3,4,5-triyltriacetate (**4o**). White solid; yield 70.3%; m. p. 188–190°C; *R*_f_ = 0.75 (ethyl acetate: petroleum ether, 1:2); IR (KBr, cm^−1^) *ν*: 3,435 (NH), 1751 (COO), 1,674 (CON); ^1^H NMR (400 MHz, CDCl_3_, ppm) *δ*: 12.45 (s, 1H, NH), 8.11 (d, *J* = 8.5 Hz, 2H, Ar-H), 7.72 (d, *J* = 8.5 Hz, 2H, Ar-H), 5.30 (t, *J* = 9.2 Hz, 1H, H-3´), 5.18–5.02 (m, 3H, H-1´, H-2´, H-4´), 4.32 (dd, *J* = 12.6, 4.8 Hz, 1H, H-5´), 4.20 (d, *J* = 12.3 Hz, 1H, H-6´), 3.82–3.79 (m, 1H, H-6´´), 2.16 (s, 3H, CH_3_), 2.13 (s, 3H, CH_3_), 2.04 (s, 3H, CH_3_), 2.01 (s, 3H, CH_3_); ^13^C NMR (150 MHz, CDCl_3_, ppm) *δ*: 170.93 (COCH_3_), 170.07 (COCH_3_), 169.34 (COCH_3_), 169.29 (COCH_3_), 164.45 (CONH), 163.34 (thiadiazole-C), 155.18 (thiadiazole-C), 132.36 (Ar-C), 130.21 (Ar-C), 129.51 (Ar-C), 128.78 (Ar-C), 83.81 (C-1´), 76.48(C-5´), 73.57 (C-3´), 69.68 (C-2´), 67.69 (C-4´), 61.69 (C-6´), 20.73 (CH_3_), 20.60 (CH_3_); HRMS [M + H]^+^ calculated for C_23_H_24_BrN_3_O_10_S_2_: m/z 646.0171, found 646.0161.

(2*R*,3*R*,4*S*,5*R*,6*R*)-2-(acetoxymethyl)-6-((5-(2-nitrobenzamido)-1,3,4-thiadiazol-2-yl)thio)tetrahy dro-2*H*-pyran-3,4,5-triyltriacetate (**4p**). Yellow solid; yield 53.4%; m. p. 188–190°C; *R*_f_ = 0.42 (ethyl acetate: petroleum ether, 1:2); IR (KBr, cm^−1^) *ν*: 3,458 (NH), 1751 (COO), 1,689 (CON); ^1^H NMR (400 MHz, CDCl_3_, ppm) *δ*: 13.02 (s, 1H, NH), 8.22 (d, *J* = 7.9 Hz, 1H, Ar-H), 7.85–7.75 (m, 3H, Ar-H), 5.32 (t, *J* = 9.0 Hz, 1H, H-3´), 5.15–5.03 (m, 3H, H-1´, H-2´, H-4´), 4.28 (dd, *J* = 12.5, 5.1 Hz, 1H, H-5´), 4.18 (dd, *J* = 12.5, 2.0 Hz, 1H, H-6´), 3.86–3.81 (m, 1H, H-6´´), 2.29 (s, 3H, CH_3_), 2.12 (s, 3H, CH_3_), 2.05 (s, 3H, CH_3_), 2.04 (s, 3H, CH_3_); ^13^C NMR (150 MHz, CDCl, ppm) *δ*: 170.97 (COCH_3_), 170.14 (COCH_3_), 169.38 (COCH_3_), 169.27 (COCH_3_), 164.63 (CONH), 162.12 (thiadiazole-C), 134.30 (thiadiazole-C), 131.80 (Ar-C), 129.81 (Ar-C), 129.50 (Ar-C), 124.88 (Ar-C), 84.14 (C-1´), 76.38 (C-5´), 73.47 (C-3´), 69.76 (C-2´), 67.73 (C-4´), 61.71 (C-6´), 20.72 (CH_3_), 20.67 (CH_3_), 20.60 (CH_3_); HRMS [M + H]^+^ calculated for C_23_H_24_N_4_O_12_S_2_: m/z 613.0915, found 613.0908.

(2*R*,3*R*,4*S*,5*R*,6*R*)-2-(acetoxymethyl)-6-((5-(4-nitrobenzamido)-1,3,4-thiadiazol-2-yl)thio)tetrahy dro-2*H*-pyran-3,4,5-triyltriacetate (**4q**). Yellow solid; yield 55.8%; m. p. 189–191°C; *R*_f_ = 0.67 (ethyl acetate: petroleum ether, 1:2); IR (KBr, cm^−1^) *ν*: 3,437 (NH), 1751 (COO), 1,678 (CON); ^1^H NMR (400 MHz, CDCl_3_, ppm) *δ*: 13.02 (s, 1H, NH), 8.48–8.42 (m, 4H, Ar-H), 5.29 (t, *J* = 9.3 Hz, 1H, H-3´), 5.19–5.12 (m, 2H, H-1´, H-2´), 4.97 (d, *J* = 10.0 Hz, 1H, H-4´), 4.35 (dd, *J* = 12.6, 4.6 Hz, 1H, H-5´), 4.26–4.22 (m, 1H, H-6´), 3.88–3.84 (m, 1H, H-6´´), 2.19 (s, 3H, CH_3_), 2.15 (s, 3H, CH_3_), 2.05 (s, 3H, CH_3_), 2.01 (s, 3H, CH_3_); ^13^C NMR (150 MHz, CDCl_3_, ppm) *δ*: 170.93 (COCH_3_), 170.03 (COCH_3_), 169.34 (COCH_3_), 169.31 (COCH_3_), 163.85 (CONH), 163.78 (thiadiazole-C), 155.13 (thiadiazole-C), 150.75 (Ar-C), 135.98 (Ar-C), 130.16 (Ar-C), 124.19 (Ar-C), 83.33 (C-1´), 76.66 (C-5´), 73.49 (C-3´),69.98 (C-2´), 67.59 (C-4´), 61.62 (C-6´), 20.72 (CH_3_), 20.69 (CH_3_), 20.60 (CH_3_), 20.58 (CH_3_), 20.57 (CH_3_); HRMS [M + H]^+^ calculated for C_23_H_24_N_4_O_12_S_2_: m/z 613.0915, found 613.0906.

### Antifungal Activity *In Vitro*

The *in vitro* antifungal activities of the target compounds against ***G*** *. zeae, Botryosphaeria dothidea* (*B. dothidea*)*, Phomopsis* sp., *P. infestans,* and *Thanatephorus cucumeris* (*T. cucumeris*) are evaluated by using the poison plate technique. All of the target compounds **4a–4q** were dissolved in 1 ml DMSO before mixing with 90 ml potato dextrose agar (PDA) to prepare concentration of 50 *μ*g/ml. Then, mycelia dishes of approximately 4 mm diameter were cut from the culture medium. A mycelium is obtained using a germ-free inoculation needle and inoculated in the middle of the PDA plate aseptically. The inoculated plates are incubated at 27 ± 1°C for 5 days. DMSO in sterile distilled water served as the negative control and Dimethomorph served as the positive control. Each treatment condition consisted of three replicates (Maddila et al., 2016). The relative inhibition rates *I* (%) were calculated as follows equation, where *C* was the diameter of fungal growth on untreated PDA, *T* was the diameter of fungi on treated PDA.

I (%) =[(C−T)/(C−0.4)]  × 100%

### Antibacterial Activity *In Vitro*

The *in vitro* antibacterial activities of the target compounds **4a–4q** against *Xoo* and *Xcc* were evaluated by using the turbidimeter test, the commercial agricultural antibacterial Thiodiazole-copper used as control. The test compounds were dissolved in 150 *μ*L of dimethylformamide (DMF) and diluted with 0.1% (v/v) Tween-20 to prepare two concentrations of 200 and 100 μg/ml. One milliliter of the liquid sample was added to the 40 ml non-toxic nutrient broth medium (NB: 1.5 g of beef extract, 2.5 g of peptone, 0.5 g of yeast powder, 5.0 g of glucose, and 500 ml of distilled water, pH 7.0–7.2). Then, 40 *μ*L of NB medium containing *Xoo* or *Xcc* was added to 5 ml of solvent NB containing the test compounds or Thiodiazole–copper. The inoculated test tubes were incubated at 30 ± 1°C under continuous shaking at 180 rpm for 48 h. The culture growth was monitored spectrophotometrically by measuring the optical density at 600 nm (OD_600_) and expressed as corrected turbidity (Dalgaard et al., 1994). The relative inhibition rates *I* (%) were calculated as follows equation, where *C*_tur_ was the corrected turbidity value of bacterial growth on untreated NB, *T*_tur_ was the corrected turbidity value of bacterial growth on treated NB.

$$I\;\left(\text{\%}\right)\;=\left(C_{\text{tur}}-T_{\text{tur}}\right)/C_{\text{tur}}\text{ × }100\text{\%}$$

## Results and Discussion

In this study, the target compounds **4a**−**4q** were synthesized in five steps, including acetylation, bromination, thioetherification, chlorination, and condensation. Among of them, it was found that 2,3,4,6-tetra-*O*-acetyl-*α*-*D*-gluco-pyranosyl bromide **1**) reacted with 2-amino-5-mercapto-1,3,4-thiadiazole to obtain (2*R*,3*R*,4*S*,5*R*,6*R*)-2-(acetoxymethyl)-6-((5-amino-1,3,4-thiadiazol-2-yl)thio)-tetrahydro-2*H*-pyran-3,4,5-triyltriacetate **2**) of *β*-configuration with high stereo selectivity in acetone solution of NaOH at room temperature, which indicated that the reaction process was S_N_2 and configuration transformation occurred in the reaction process.

All the synthesized compounds were characterized by ^1^H NMR, ^13^C NMR, and HRMS. In the ^1^H NMR spectra of the obtained amide, pyran and acetyl proton signals should be distinguished. For example, for compound **4i**, the proton signals of NH group was observed as a singlet at 12.54 ppm, signals of benzene ring protons were registered at 8.36 and 7.85 ppm, respectively, and the proton signal of pyran was registered in the range of 5.18–3.82 ppm. Moreover, four singlets at 2.17, 2.09, 2.04, and 2.01 ppm indicated to CH_3_ protons of acetyl.

The *in vitro* antifungal activities of the target compounds were evaluated against five different fungus including *P. infestans,* ***G*** *. zeae,* ***B*** *. dothidea, Phomopsis* sp., and *T. cucumeris*. Bioassay results, as shown in Table 1, revealed that the target compounds exhibited moderate to good antifungal activities against *P. infestans,* ***G*** *. zeae,* ***B*** *. dothidea, Phomopsis* sp., and *T. cucumeris*, with the inhibitory rates range of 19.8–83.5%, 35.6–73.1%, 22.1–62.0%, 21.0–64.0%, and 17.1–65.1%, respectively. Meanwhile, it was found that the inhibitory rates of the target compounds against ***G*** *. zeae* in the range of 35.6–73.1% at the 50 μg/ml, which was higher than the previously reported inhibitory activity of *N*-(2-chloro-4-phenyl-5-(trifluoromethyl)cyclopenta-1,4-dien-1-yl)-5-((4-nitrobenzyl)thio)-1,3,4-thiadiazol-2-amine against ***G*** *. zeae* (23.9%) at the 50 μg/ml (Xie et al., 2016). Especially, compound **4i** and **4q** showed higher antifungal activity against *P. infestans,* with the inhibition rates of 83.5%, 81.1%, respectively, than that of Dimethomorph (78.2%). Based on the preliminary antifungal bioassays, the EC_50_ values of partial compounds against *P. infestans* were also tested and presented in Table 2. Table 2 showed that compounds **4i** exhibited good bioactivities against *P. infestans*, with EC_50_ values of 3.43 μg/ml, which were higher than that of Dimethomorph (5.52 μg/ml). While, the target compounds showed lower antibacterial activities (Table 3) against *Xoo* and *Xcc* at 200 and 100 μg/ml than those of Thiodiazole-copper as well as the amide derivatives containing 1,3,4-thiadiazole of the previously reported by Chen (Chen J. et al., 2019).

**TABLE 1:** The *in vitro* antifungal activities of the target compounds **4a–4q** at 50 μg/ml.

Compounds	Inhibition rate (%)				
	*G. zeae*	*B. dothidea*	*P. infestans*	*Phomopsis* sp	*T. cucumeris*
**4a**	58.6 ± 2.2	58.1 ± 1.6	44.4 ± 1.5	21.0 ± 2.4	17.1 ± 1.2
**4b**	62.2 ± 1.4	54.8 ± 0.7	28.5 ± 2.0	38.7 ± 1.3	29.0 ± 1.2
**4c**	65.7 ± 1.3	60.1 ± 1.1	19.8 ± 0.6	43.0 ± 2.9	56.9 ± 2.4
**4days**	58.9 ± 1.1	52.0 ± 1.2	40.9 ± 1.4	50.0 ± 1.3	44.5 ± 1.5
**4e**	53.6 ± 0.7	40.7 ± 1.1	29.4 ± 0.7	26.7 ± 0.4	32.0 ± 1.4
**4f**	51.7 ± 1.1	43.3 ± 0.1	35.0 ± 1.9	30.8 ± 2.3	42.2 ± 2.0
**4g**	58.4 ± 1.2	60.7 ± 1.2	77.3 ± 2.1	56.7 ± 2.1	62.0 ± 1.0
**4h**	35.6 ± 0.6	33.5 ± 0.8	73.0 ± 1.0	30.8 ± 1.0	22.2 ± 2.2
**4i**	48.9 ± 1.7	58.1 ± 1.5	83.5 ± 0.6	55.2 ± 2.1	64.3 ± 1.5
**4j**	58.3 ± 1.6	51.1 ± 0.9	30.1 ± 2.6	58.4 ± 1.7	44.7 ± 1.6
**4k**	55.2 ± 2.2	55.2 ± 1.2	61.9 ± 2.0	43.7 ± 2.0	37.0 ± 1.8
**4L**	58.0 ± 2.3	49.2 ± 1.3	70.0 ± 1.2	31.5 ± 0.9	59.8 ± 0.9
**4m**	73.1 ± 1.0	41.0 ± 1.6	63.6 ± 1.3	48.4 ± 1.1	44.3 ± 1.6
**4n**	70.3 ± 1.1	45.6 ± 1.1	73.1 ± 1.8	33.7 ± 0.8	58.5 ± 1.8
**4o**	45.0 ± 2.2	22.1 ± 0.9	75.9 ± 1.2	40.0 ± 2.3	54.3 ± 1.7
**4p**	53.4 ± 1.9	61.3 ± 1.1	79.0 ± 1.1	64.0 ± 1.3	62.8 ± 0.7
**4q**	56.8 ± 1.5	62.0 ± 2.0	81.1 ± 0.3	63.1 ± 1.2	65.1 ± 1.3
Dimethomorph	74.3 ± 2.0	72.3 ± 1.6	78.2 ± 1.1	69.3 ± 1.6	68.3 ± 1.6

**TABLE 2:** The EC_50_ values of compounds **4i, 4p,** and **4q** against *P. infestans.*

Compds	Toxic regression equation	*r*	EC_50_ (μg/ml)
**4i**	y = 0.85x + 4.53	0.98	3.43 ± 1.5
**4p**	y = 0.98x + 4.22	0.98	6.15 ± 2.1
**4q**	y = 1.13x + 4.20	0.97	5.02 ± 1.8
Dimethomorph	y = 0.94x + 4.30	0.99	5.52 ± 1.2

**TABLE 3:** The *in vitro* antibacterial activities of the target compounds **4a–4q**.

Compds	*Xoo*		*Xcc*	
	200 μg/ml	100 μg/ml	200 μg/ml	100 μg/ml
**4a**	60.1 ± 1.1	38.1 ± 2.1	64.9 ± 1.2	31.7 ± 2.2
**4b**	63.5 ± 1.5	37.3 ± 1.3	60.1 ± 2.2	39.2 ± 1.4
**4c**	54.2 ± 2.0	38.5 ± 1.0	55.4 ± 1.9	34.8 ± 2.1
**4days**	58.6 ± 1.8	42.3 ± 1.3	66.8 ± 2.1	36.3 ± 2.8
**4e**	44.0 ± 2.1	35.2 ± 1.5	51.4 ± 1.5	34.9 ± 2.2
**4f**	43.6 ± 1.9	32.6 ± 1.6	47.3 ± 1.5	25.8 ± 1.7
**4g**	49.0 ± 1.5	31.7 ± 2.3	33.2 ± 1.9	16.6 ± 1.5
**4h**	45.2 ± 1.5	33.4 ± 2.1	67.2 ± 2.0	43.3 ± 2.6
**4i**	59.4 ± 2.2	34.4 ± 1.7	68.6 ± 1.0	39.6 ± 1.4
**4j**	53.5 ± 1.6	32.8 ± 1.3	61.9 ± 1.3	45.5 ± 2.1
**4k**	51.0 ± 1.6	31.6 ± 1.1	26.5 ± 1.8	15.6 ± 1.7
**4L**	71.2 ± 0.9	42.6 ± 1.0	77.5 ± 1.4	45.3 ± 2.6
**4m**	74.4 ± 1.2	44.8 ± 1.5	77.5 ± 1.6	42.3 ± 1.6
**4n**	68.4 ± 2.1	42.6 ± 1.1	79.0 ± 2.0	47.2 ± 1.8
**4o**	74.6 ± 1.6	43.8 ± 1.3	75.8 ± 2.8	45.1 ± 1.3
**4p**	70.1 ± 2.5	43.1 ± 1.4	76.2 ± 2.0	43.1 ± 1.2
**4q**	69.7 ± 1.2	42.3 ± 1.4	80.8 ± 2.5	45.0 ± 1.3
Thiodiazole-copper	76.2 ± 1.3	45.2 ± 1.3	86.2 ± 2.1	44.5 ± 1.7

From the structure-activity relationships (SAR) analysis, it was found that there was clear SAR against *P. infestans*. Inspection of the chemical structures of the target compounds suggests that the group R in the target compounds significantly influence the antifungal activity against *P. infestans*. With a fluorinated or nitrificated substituent (4-F and 4-NO_2_) on the phenyl ring, the compounds exhibited enhanced bioactivity against *P. infestans* (**4i** and **4q**). Further, the position of substituent groups in the phenyl ring also plays an important role in the antifungal activity against *P. infestans*, with a four substituent (4-F or 4-NO_2_) in the phenyl ring exhibited higher antifungal activity than other positions.

## Conclusion

A series of novel 1,3,4-thiadiazole derivatives of glucosides were prepared via acetylation, bromination, thioetherification, chlorination, and condensation. Bioassay results showed that some of the target compounds revealed better inhibitory activity against *P. infestans*. In addition, SAR analysis found that the type and position of substituent groups in the phenyl ring of the target compounds plays an important role in increasing the antifungal activity against *P. infestans*.

## Data Availability

The original contributions presented in the study are included in the article/Supplementary Material; further inquiries can be directed to the corresponding author/s.

## References

[B1] Aggarwal N. Kumar R. Dureja P. Khurana J. M. (2012). Synthesis of novel nalidixic acid-based 1,3,4-thiadiazole and 1,3,4-oxadiazole derivatives as potent antibacterial agents. Chem. Biol. Drug Des. 79, 384–397. 10.1111/j.1747-0285.2011.01316.x 22212247

[B2] Aktar M. W. Sengupta D. Chowdhury A. (2009). Impact of pesticides use in agriculture: their benefits and hazards. Interdiscipl. Toxicol. 2, 1–12. 10.2478/v10102-009-0001-7 PMC2984095 21217838

[B3] Alwan W. S. Karpoormath R. Palkar M. B. Patel H. M. Rane R. A. Shaikh M. S. (2015). Novel imidazo[2,1-*b*]-1,3,4-thiadiazole as promising antifungal agents against clinical isolate of *Cryptococcus neoformans* . Eur. J. Med. Chem. 95, 514–525. 10.1016/j.ejmech.2015.03.02110.1016/j.ejmech.2015.03.021 25847769

[B4] Bhinge S. D. Chature V. Sonawane L. V. (2015). Synthesis of some novel 1,3,4-thiadiazole derivatives and biological screening for anti-microbial, antifungal and anthelmintic activity. Pharm. Chem. J. 49, 1–6. 10.1007/s11094-015-1287-8

[B5] Chen J. Yi C. Wang S. Wu S. Li S. Hu D. (2019). Novel amide derivatives containing 1,3,4-thiadiazole moiety: design, synthesis, nematocidal and antibacterial activities. Bioorg. Med. Chem. Lett. 29, 1203–1210. 10.1016/j.bmcl.2019.03.01710.1016/j.bmcl.2019.03.017 30902458

[B6] Chen W. Zhang H. Wang J. Hu X. (2019). Flavonoid glycosides from the bulbs of *lilium speciosum* var*. gloriosoides* and their potential antiviral activity against. RSV. Chem. Nat. Compd. 55, 461–464. 10.1007/s10600-019-02714-7

[B7] Chudzik B. Bonio K. Dabrowski W. Pietrzak D. Niewiadomy A. Olender A. (2019). Synergistic antifungal interactions of amphotericin B with 4-(5-methyl-1,3,4-thiadiazole-2-yl) benzene-1,3-diol. Sci. Rep. 9, 12945–12959. 10.1038/s41598-019-49425-1 31506532 PMC6737028

[B8] Dalgaard P. Ross T. Kamperman L. Neumeyer K. Mcmeekin T. A. (1994). Estimation of bacterial growth rates from turbidimetric and viable count data. Int. J. Food Microbiol. 23, 391–404. 10.1016/0168-1605(94)90165-1 7873339

[B30] Gruner S. A. W. Locardi E. Lohof E. Kessler H. (2009). Carbohydrate-based mimetics in drug design: sugar amino acids and carbohydrate scaffolds. Chem. Rev. 102, 491–456. 10.1021/cr0004409 11841252

[B9] Gurung R. B. Gong S. Y. Dhakal D. Le T. T. Jung N. R. Hye j. j (2018). Synthesis of curcumin glycosides with enhanced anticancer properties using one-pot multienzyme glycosylation technique. J. Microbiol. Biotechnol. 27, 1639–1648. 10.4014/jmb.1701.01054 28633516

[B10] Hawas U. W. El-Kassem L. T. A. Shaher F. Al-Farawati R. (2019). *In vitro* inhibition of hepatitis c virus protease and antioxidant by flavonoid glycosides from the saudi costal plant *sarcocornia fruticosa* . Nat. Prod. Lett. 33, 3364–3371. 10.1080/14786419.2018.1477153 29897265

[B11] He X. Wang Y. Luo R. H. Yang L. M. Wang L.,Dale, G. (2019). Dimeric pyranonaphthoquinone glycosides with anti-HIV and cytotoxic activities from a*soil-derived streptomyces* . J. Nat. Prod. 82, 1813–1819. 10.1021/acs.jnatprod.9b00022 31310115

[B12] Hu H. Z. Xiang G. X. Chen J. X. Chen W. Wu l. Xu S. (1997). An antibiotic pesticide‐Ningnanmycin, CN1036307C.

[B13] Jiang X. L. Wang L. Wang E. J. Zhang G. L. Chen B. Wang M. K. (2018). Flavonoid glycosides and alkaloids from the embryos of *nelumbo nucifera* seeds and their antioxidant activity. Fitoterapia. 125, 184–190. 10.1016/j.fitote.2018.01.009 29371159

[B14] Kamat M. N. Rath N. P. Demchenko A. V. (2007). Versatile synthesis and mechanism of activation of *s*-benzoxazolyl glycosides. J. Org. Chem. 72 (18), 6938–6946. 10.1021/jo.071184410.1021/jo0711844 17676918 PMC2535574

[B15] Khodair A. I. Attia A. M. Gendy E. A. Elshaier Y. A. M. M. MohammedEl‐Magd A. M. A. (2019). Discovery of new *S*‐glycosides and *N*‐glycosides of pyridine‐biphenyl system with antiviral activity and induction of apoptosis in MCF7 cells. J. Heterocycl. Chem. 56, 1733–1746. 10.1002/jhet.3527

[B16] Knyazyan A. Eliazyan K. Pivazyan V. Ghazaryan E. Harutyunyan S. Yengoyan A. (2012). Synthesis and growth regulatory activity of novel 5-(3-alkyl-4-methyl-2-thioxo-2,3-dihydro-thiazol-5-yl)-3*H*-[1,3,4]thiadiazole(oxadiazole)-2-thiones and their derivatives. Heterocycl. Commun. 18, 103–108. 10.1515/hc-2012-0040

[B17] Maddila S. Gorle S. Sampath C. Lavanya P. (2016). Synthesis and anti-inflammatory activity of some new 1,3,4-thiadiazole containing pyrazole and pyrrole nucleus. J. Saudi Chem. Soc. 20, 306–312. 10.1016/j.jscs.2012.11.007

[B18] Mohammed H. S. Abdel-Aziz M. M. Abu-Baker M. S. Saad A. M. Mohamed M. A. Ghareeb M. A. (2019). Antibacterial and potential antidiabetic activities of flavone *C*-glycosides isolated from *Beta vulgaris* subspecies cicla. var. flavescens (Amaranthaceae) cultivated in Egypt. Curr. Pharmaceut. Biotechnol. 20, 595–604. 10.2174/1389201020666190613161212 31203800

[B19] Rahim A. Mostofa M. G. Sadik M. G. Rahman M. A. A. Alam A. K. (2020). The anticancer activity of two glycosides from the leaves of *Leea aequata* L. Nat. Prod. Res. (27). 1–5. 10.1080/14786419.2020.1798661 32713195

[B20] Scattolin T. Bortolamiol E. Rizzolio F. Demitri N. Visentin F. (2020). Allyl palladium complexes bearing carbohydrate-based n-heterocyclic carbenes: anticancer agents for selective and potent *in vitro* cytotoxicity. Appl. Organomet. Chem. 34, e5876. 10.1002/aoc.5876

[B21] Sridhar G. Palle S. Vantikommu J. Gangarapu K. (2020). Design, synthesis, and biological evaluation of amide derivatives of imidazo[2,1-*b*][1,3,4]thiadiazole as anticancer agents. Synth. Commun. 50, 3221–3233. 10.1080/00397911.2020.1797814

[B22] Taflan E. Bayrak H. Er M. Alpay Karaoğlu Ş. Bozdeveci A. (2019). Novel imidazo[2,1-*b*][1,3,4]thiadiazole (ITD) hybrid compounds: design, synthesis, efficient antibacterial activity and antioxidant effects. Bioorg. Chem. 89, 102998. 10.1016/j.bioorg.2019.102998 31128819

[B23] Wu M. Han G. Meng C. Wang Z. Liu Y. Wang Q. (2014). Design, synthesis, and anti-tobacco mosaic virus (TMV) activity of glycoconjugates of phenanthroindolizidines alkaloids. Mol. Divers. 18, 25–37. 10.1007/s11030-013-9484-4 24132551

[B24] Xie Y. Gong H. Wang X. Ruan X. Zhang J. Li Q. (2016). Synthesis and biological activity of novel pyrazole amide derivatives containing 1,3,4-thiadiazole thioether moiety. Agrochemicals. 55, 872–876.

[B25] Yang X. H. Xiang L. Li X. Zhao T. T. Zhang H. Zhou W. P. (2012). Synthesis, biological evaluation, and molecular docking studies of 1,3,4-thiadiazol-2-amide derivatives as novel anticancer agents. Bioorg. Med. Chem. 20, 2789–2795. 10.1016/j.bmc.2012.03.040 22503364

[B26] Zhan J. Thrall P. H. Papaïx J. Xie L. Burdon J. J. (2015). Playing on a pathogen’s weakness: using evolution to guide sustainable plant disease control strategies. Annu. Rev. Phytopathol. 53, 19–43. 10.1146/annurev-phyto-080614-120040 25938275

[B28] Zhang M. Xu W. Wei K. Liu H. Xue W. (2019). Synthesis and evaluation of 1,3,4‐thiadiazole derivatives containing cyclopentylpropionamide as potential antibacterial agent. J. Heterocycl. Chem. 56, 1966–1977. 10.1002/jhet.3576

