# Supplementary material for: Synthesis and Bioactivities of Novel 1,3,4-Thiadiazole Derivatives of Glucosides
Source: Front Chem. 2021 Mar 26;9:645876. doi: 10.3389/fchem.2021.645876 (PMC8032861; doi:10.3389/fchem.2021.645876)
Supplement: Supplementary file 2 [file datasheet2.pdf]

TABLE 2. The EC_{50} values of compounds **4i, 4p,** and **4q** against *P. infestans.*

Compds.	Toxic regression equation	*r*	EC_{50} (μg/mL)
**4i**	y = 0.85x + 4.53	0.98	3.43
**4p**	y = 0.98x + 4.22	0.98	6.15
**4q**	y = 1.13x + 4.20	0.97	5.02
Dimethomorph	y = 0.94x + 4.30	0.99	5.52
