# Supplementary material for: Synthesis and Bioactivities of Novel 1,3,4-Thiadiazole Derivatives of Glucosides
Source: Front Chem. 2021 Mar 26;9:645876. doi: 10.3389/fchem.2021.645876 (PMC8032861; doi:10.3389/fchem.2021.645876)
Supplement: Supplementary file 3 [file datasheet3.pdf]

TABLE 1. The *in vitro* antifungal activities of the target compounds **4a–4q** at 50 μg/mL.

**Compounds**	Inhibition rate (%)				
	G. zeae	*B. dothidea*	*P. infestans*	*Phompsis* sp.	*T. cucumeris*
**4a**	58.6 ± 2.2	58.1 ± 1.6	44.4 ± 1.5	21.0 ± 2.4	17.1 ± 1.2
**4b**	62.2 ± 1.4	54.8 ± 0.7	28.5 ± 2.0	38.7 ± 1.3	29.0 ± 1.2
**4c**	65.7 ± 1.3	60.1 ± 1.1	19.8 ± 0.6	43.0 ± 2.9	56.9 ± 2.4
**4d**	58.9 ± 1.1	52.0 ± 1.2	40.9 ± 1.4	50.0 ± 1.3	44.5 ± 1.5
**4e**	53.6 ± 0.7	40.7 ± 1.1	29.4 ± 0.7	26.7 ± 0.4	32.0 ± 1.4
**4f**	51.7 ± 1.1	43.3 ± 0.1	35.0 ± 1.9	30.8 ± 2.3	42.2 ± 2.0
**4g**	58.4 ± 1.2	60.7 ± 1.2	77.3 ± 2.1	56.7 ± 2.1	62.0 ± 1.0
**4h**	35.6 ± 0.6	33.5 ± 0.8	73.0 ± 1.0	30.8 ± 1.0	22.2 ± 2.2
**4i**	48.9 ± 1.7	58.1 ± 1.5	83.5 ± 0.6	55.2 ± 2.1	64.3 ± 1.5
**4j**	58.3 ± 1.6	51.1 ± 0.9	30.1 ± 2.6	58.4 ± 1.7	44.7 ± 1.6
**4k**	55.2 ± 2.2	55.2 ± 1.2	61.9 ± 2.0	43.7 ± 2.0	37.0 ± 1.8
**4l**	58.0 ± 2.3	49.2 ± 1.3	70.0 ± 1.2	31.5 ± 0.9	59.8 ± 0.9
**4m**	73.1 ± 1.0	41.0 ± 1.6	63.6 ± 1.3	48.4 ± 1.1	44.3 ± 1.6
**4n**	70.3 ± 1.1	45.6 ± 1.1	73.1 ± 1.8	33.7 ± 0.8	58.5 ± 1.8
**4o**	45.0 ± 2.2	22.1 ± 0.9	75.9 ± 1.2	40.0 ± 2.3	54.3 ± 1.7
**4p**	53.4 ± 1.9	61.3 ± 1.1	79.0 ± 1.1	64.0 ± 1.3	62.8 ± 0.7
**4q**	56.8 ± 1.5	62.0 ± 2.0	81.1 ± 0.3	63.1 ± 1.2	65.1 ± 1.3
Dimethomorph	74.3 ± 2.0	72.3 ± 1.6	78.2 ± 1.1	69.3 ± 1.6	68.3 ± 1.6

TABLE 2. The EC_{50} values of compounds **4i, 4p,** and **4q** against *P. infestans.*

Compds.	Toxic regression equation	*r*	EC_{50} (μg/mL)
**4i**	y = 0.85x + 4.53	0.98	3.43
**4p**	y = 0.98x + 4.22	0.98	6.15
**4q**	y = 1.13x + 4.20	0.97	5.02
Dimethomorph	y = 0.94x + 4.30	0.99	5.52

TABLE 3. The *in vitro* antibacterial activities of the target compounds **4a–4q**.

**Compds.**	*Xoo*		*Xcc*	
	200 μg/mL	100 μg/mL	200 μg/mL	100 μg/mL

**4a**	60.1 ± 1.1	38.1 ± 2.1	64.9 ± 1.2	31.7 ± 2.2
**4b**	63.5 ± 1.5	37.3 ± 1.3	60.1 ± 2.2	39.2 ± 1.4
**4c**	54.2 ± 2.0	38.5 ± 1.0	55.4 ± 1.9	34.8 ± 2.1
**4d**	58.6 ± 1.8	42.3 ± 1.3	66.8 ± 2.1	36.3 ± 2.8
**4e**	44.0 ± 2.1	35.2 ± 1.5	51.4 ± 1.5	34.9 ± 2.2
**4f**	43.6 ± 1.9	32.6 ± 1.6	47.3 ± 1.5	25.8 ± 1.7
**4g**	49.0 ± 1.5	31.7 ± 2.3	33.2 ± 1.9	16.6 ± 1.5
**4h**	45.2 ± 1.5	33.4 ± 2.1	67.2 ± 2.0	43.3 ± 2.6
**4i**	59.4 ± 2.2	34.4 ± 1.7	68.6 ± 1.0	39.6 ± 1.4
**4j**	53.5 ± 1.6	32.8 ± 1.3	61.9 ± 1.3	45.5 ± 2.1
**4k**	51.0 ± 1.6	31.6 ± 1.1	26.5 ± 1.8	15.6 ± 1.7
**4l**	71.2 ± 0.9	42.6 ± 1.0	77.5 ± 1.4	45.3 ± 2.6
**4m**	74.4 ± 1.2	44.8 ± 1.5	77.5 ± 1.6	42.3 ± 1.6
**4n**	68.4 ± 2.1	42.6 ± 1.1	79.0 ± 2.0	47.2 ± 1.8
**4o**	74.6 ± 1.6	43.8 ± 1.3	75.8 ± 2.8	45.1 ± 1.3
**4p**	70.1 ± 2.5	43.1 ± 1.4	76.2 ± 2.0	43.1 ± 1.2
**4q**	69.7 ± 1.2	42.3 ± 1.4	80.8 ± 2.5	45.0 ± 1.3
Thiodiazole-copper	76.2 ± 1.3	45.2 ± 1.3	86.2 ± 2.1	44.5 ± 1.7
