# Supplementary material for: Synthesis and Bioactivities of Novel 1,3,4-Thiadiazole Derivatives of Glucosides
Source: Front Chem. 2021 Mar 26;9:645876. doi: 10.3389/fchem.2021.645876 (PMC8032861; doi:10.3389/fchem.2021.645876)
Supplement: Supplementary file 4 [file table1.docx]

# *Supplementary Material*

# Data of ^1^H NMR spectrum of the intermediate 1 and 2

2,3,4,6-tetra-*O*-acetyl-*α*-*D*-glucopyranosyl bromide (**1**): ^1^H NMR (400 MHz, CDCl_3_, ppm) *δ*: 6.55 (d, *J* = 4.0 Hz, 1H，H-1´), 5.49 (t, *J* = 9.7 Hz, 1H, H-2´), 5.10 (t, *J* = 9.8 Hz, 1H, H-3´), 4.77 (dd, *J* = 10.0, 4.0 Hz, 1H, H-4´), 4.28 – 4.22 (m, 2H, H-5´, H-6´), 4.06 (d, *J* = 10.8 Hz, 1H, H-6´´), 2.04 (s, 3H, CH_3_), 2.03 (s, 3H, CH_3_), 1.99 (s, 3H, CH_3_), 1.97 (s, 3H, CH_3_).


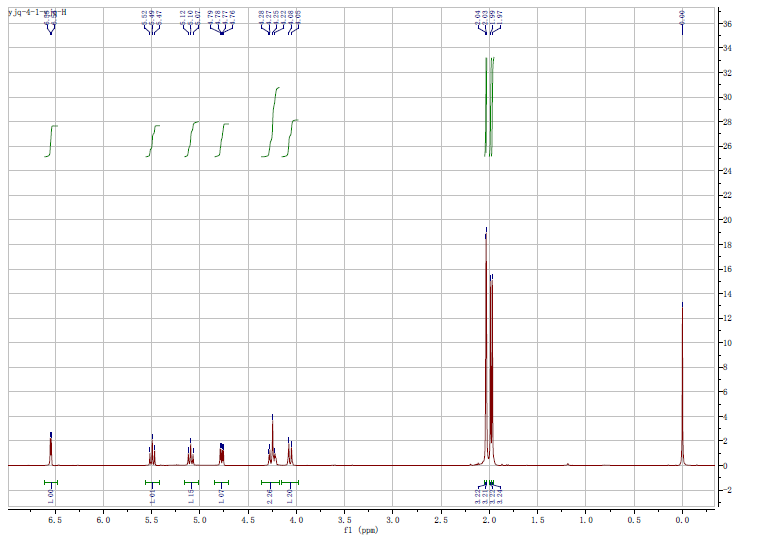


^1^H NMR spectrum of the intermediate **1**

(2R,3R,4S,5R,6R)-2-(acetoxymethyl)-6-((5-amino-1,3,4-thiadiazol-2-yl)thio)tetrahydro-2*H*-pyran-3,4,5-triyl triacetate (**2**): ^1^H NMR (600 MHz, DMSO-*d*_6,_ ppm) *δ*: 7.50 (s, 2H, NH_2_), 5.39 (t, *J* = 9.4 Hz, 1H, H-1´), 5.32 (d, *J* = 10.0 Hz, 1H, H-2´), 4.96 (t, *J* = 9.7 Hz, 1H, H-3´), 4.90 (t, *J* = 9.7 Hz, 1H, H-4´), 4.18 (dd, *J* = 12.1, 5.4 Hz, 1H, H-5´), 4.14 – 4.05 (m, 2H, , H-6´, H-6´´), 2.05 (s, 6H, 2CH_3_), 2.00 (s, 3H, CH_3_), 1.95 (s, 3H, CH_3_).

^1^H NMR spectrum of the intermediate **2**

# 2. Figures of ^1^H NMR, ^13^C NMR and HRMS spectrum of the target compounds (4a-4q)

IR spectrum of the target compounds **4a**

^
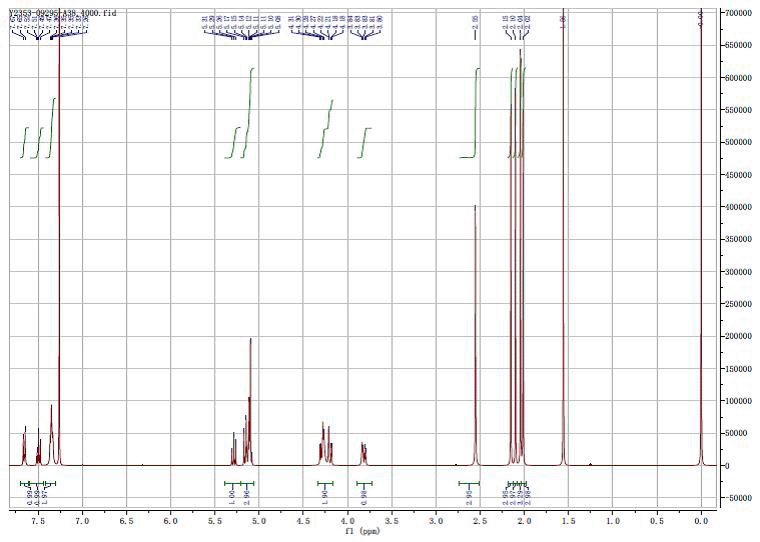
^

^1^H NMR spectrum of the target compounds **4a**


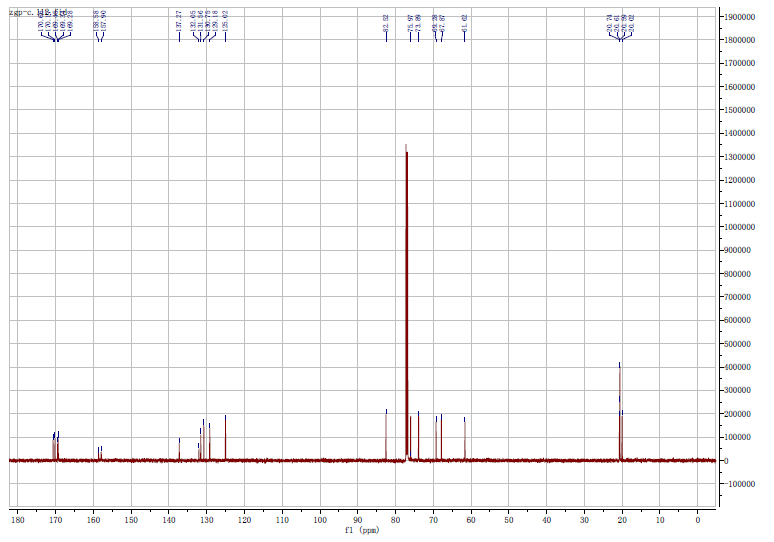


^13^C NMR spectrum of the target compounds **4a**


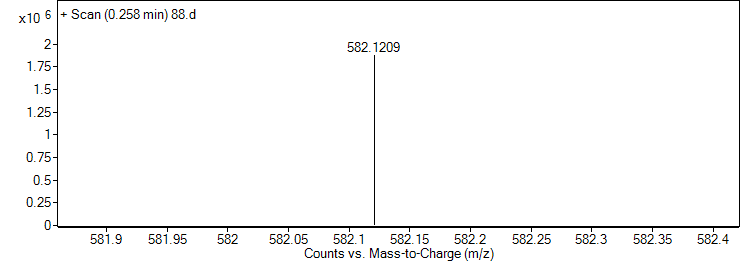


HRMS spectrum of the target compounds **4a**

IR spectrum of the target compounds **4b**

^1^H NMR spectrum of the target compounds **4b**

**
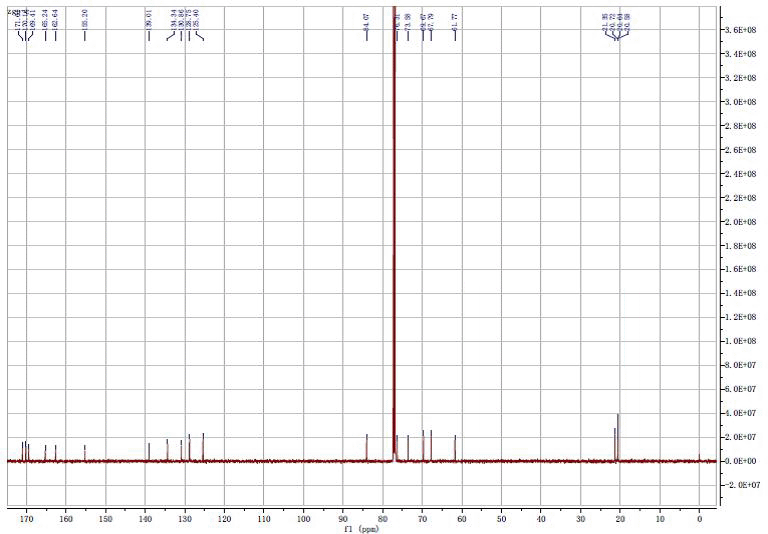
**

^13^C NMR spectrum of the target compounds **4b**

**
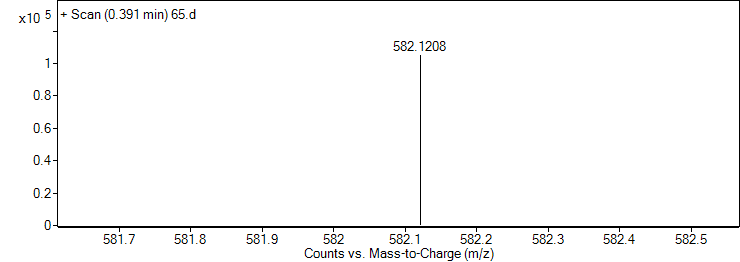
**

HRMS spectrum of the target compounds **4b**

IR spectrum of the target compounds **4c**


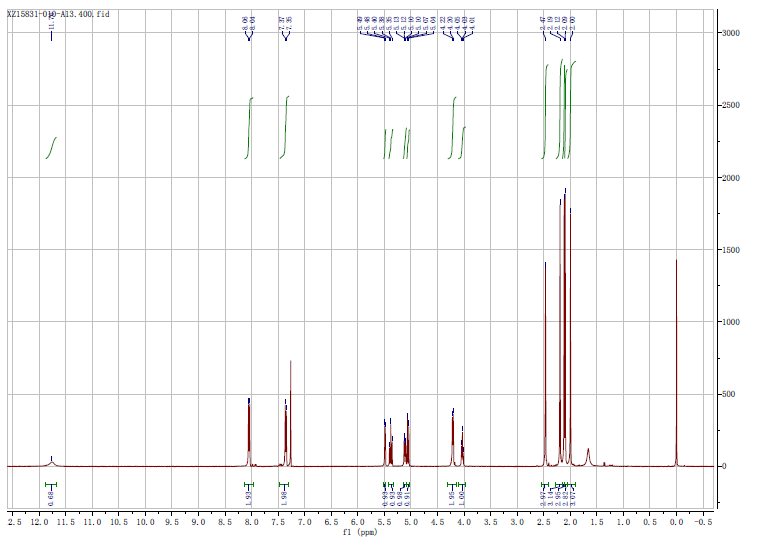


^1^H NMR spectrum of the target compounds **4c**

**
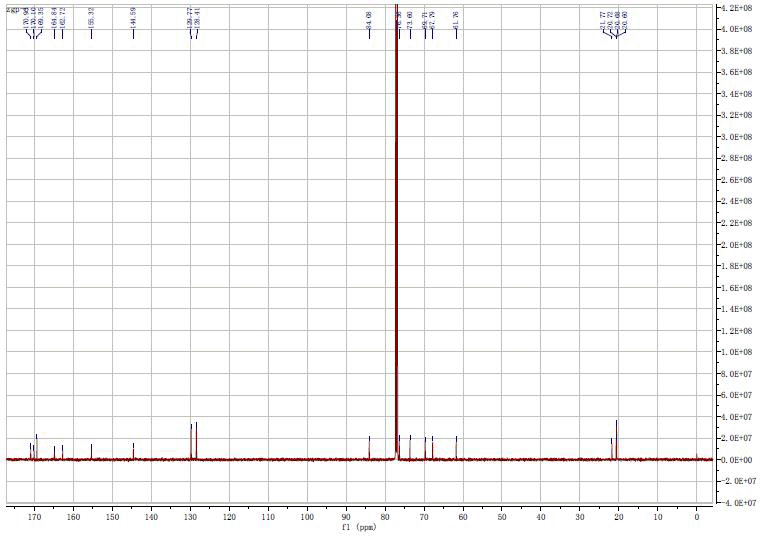
**

^13^C NMR spectrum of the target compounds **4c**

**
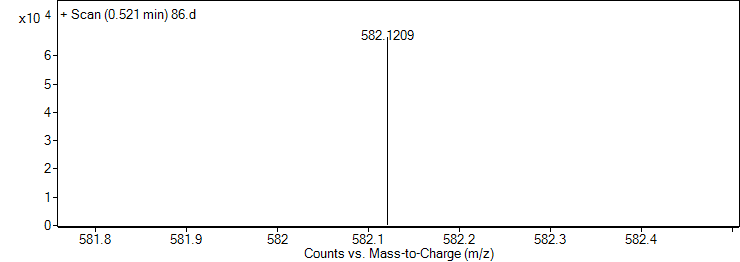
**

HRMS spectrum of the target compounds **4c**

IR spectrum of the target compounds **4d**


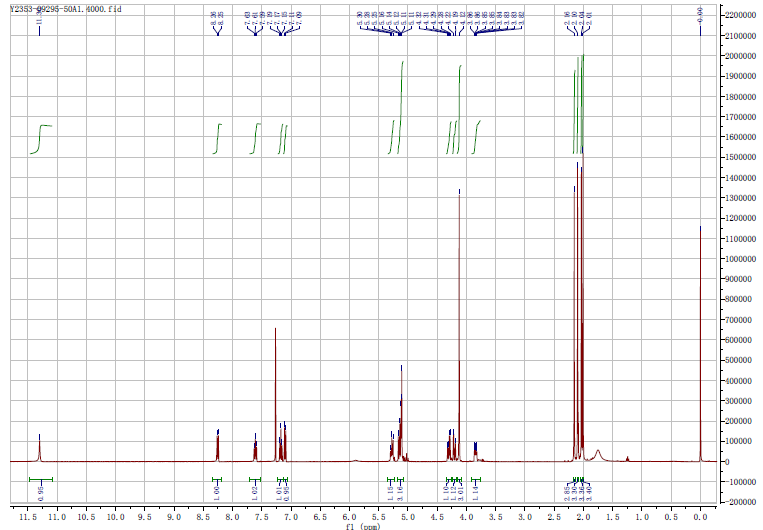


^1^H NMR spectrum of the target compounds **4d**

^^

^13^C NMR spectrum of the target compounds **4d**

**
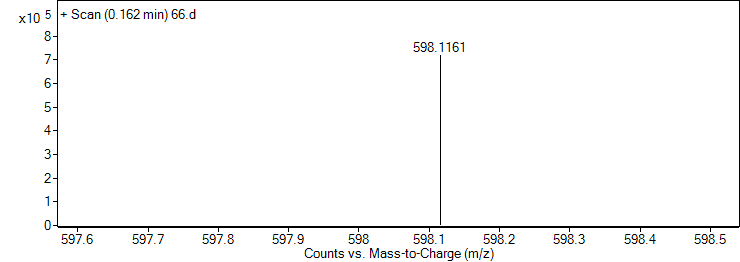
**

HRMS spectrum of the target compounds **4d**

IR spectrum of the target compounds **4e**

**
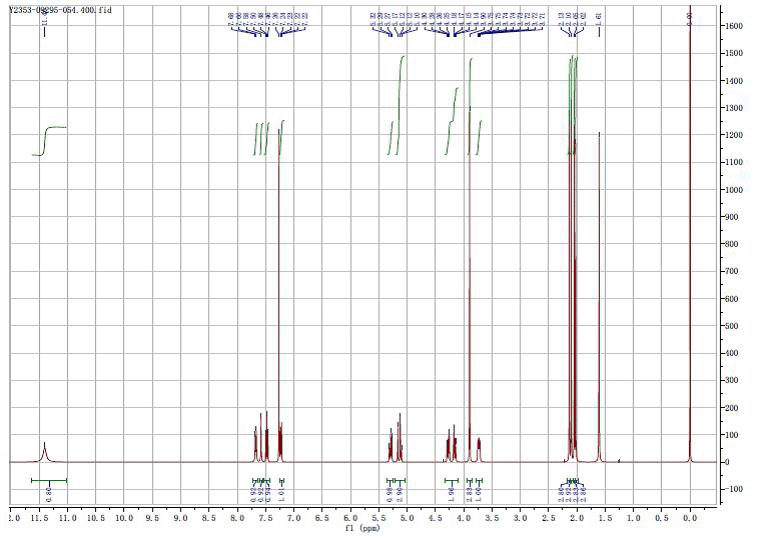
**

^1^H NMR spectrum of the target compounds **4e**

^13^C NMR spectrum of the target compounds **4e**


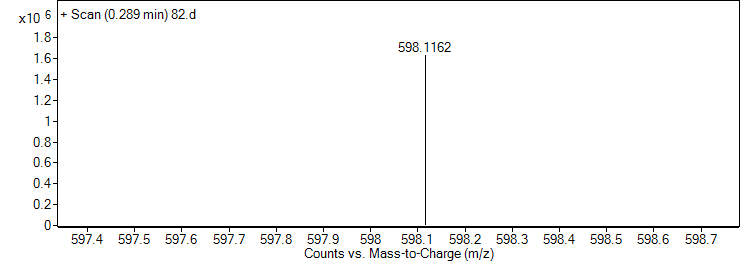
HRMS spectrum of the target compounds **4e**

IR spectrum of the target compounds **4f**

**
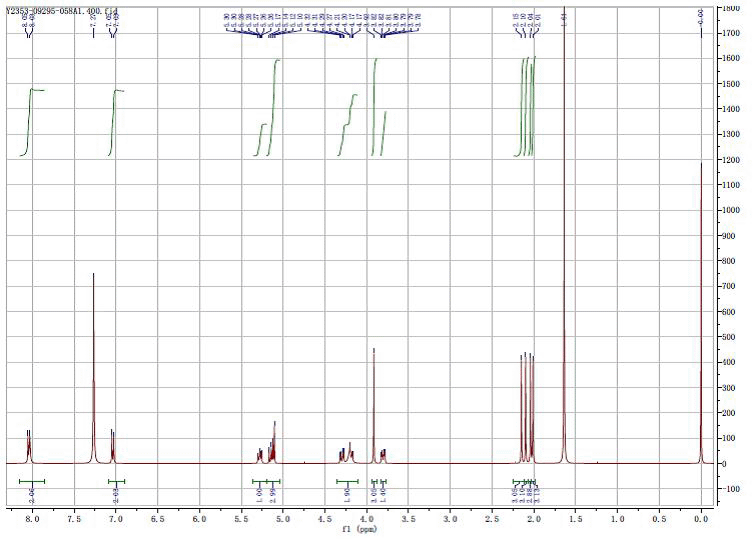
**

^1^H NMR spectrum of the target compounds **4f**

**
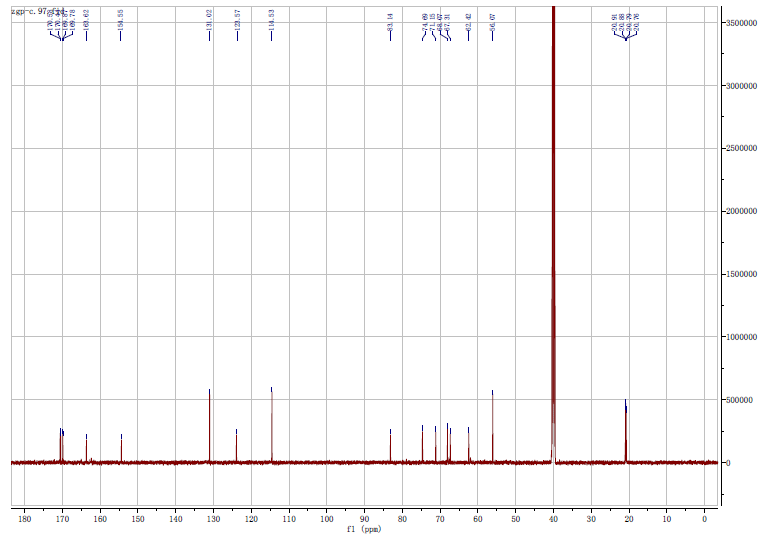
**

^13^C NMR spectrum of the target compounds **4f**

**
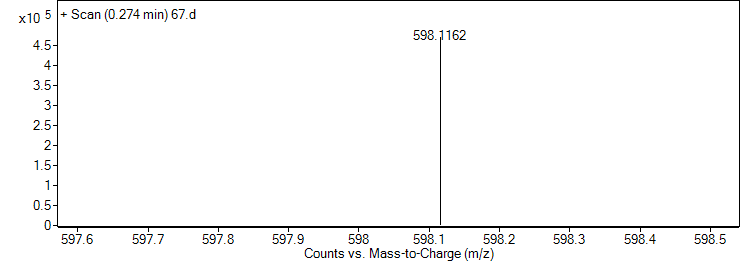
**

HRMS spectrum of the target compounds **4f**

IR spectrum of the target compounds **4g**

**

**

^1^H NMR spectrum of the target compounds **4g**

^13^C NMR spectrum of the target compounds **4g**

**
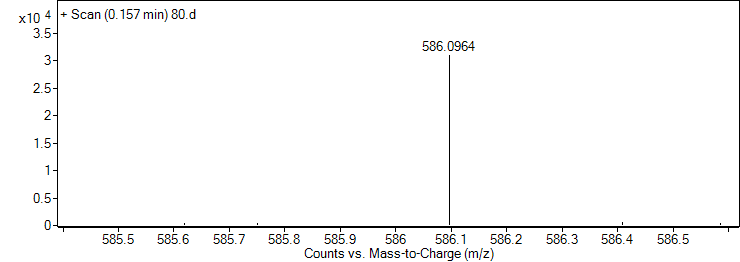
**

HRMS spectrum of the target compounds **4g**

IR spectrum of the target compounds **4h**

^1^H NMR spectrum of the target compounds **4h**


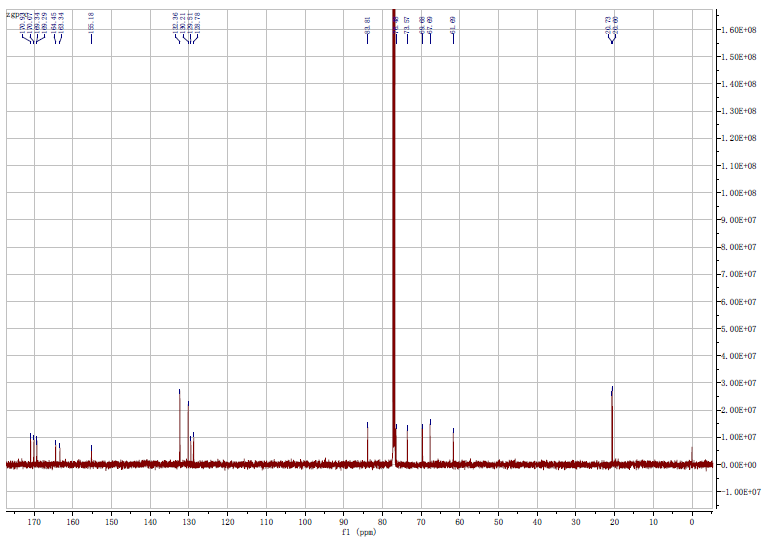


^13^C NMR spectrum of the target compounds **4h**


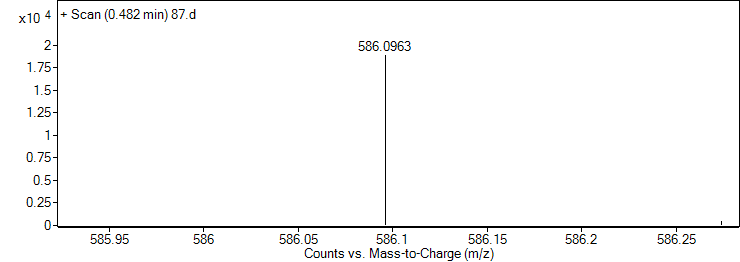
HRMS spectrum of the target compounds **4h**

IR spectrum of the target compounds **4i**

**
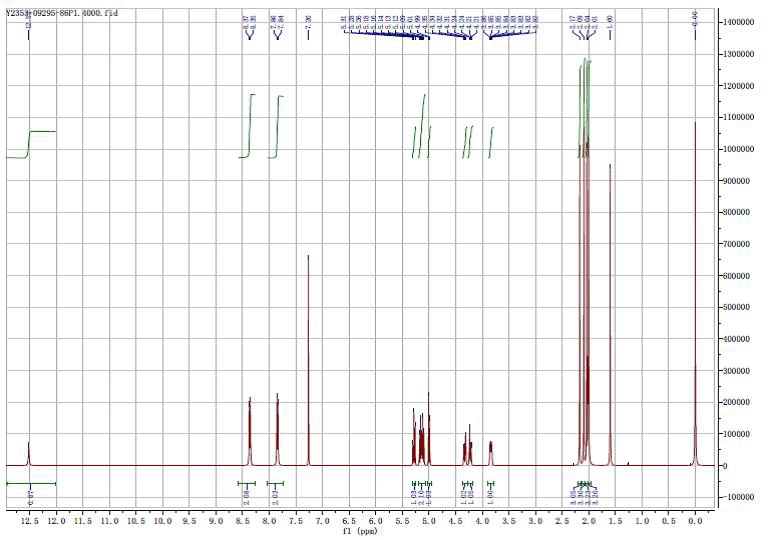
**

^1^H NMR spectrum of the target compounds **4i**

^13^C NMR spectrum of the target compounds **4i**

**
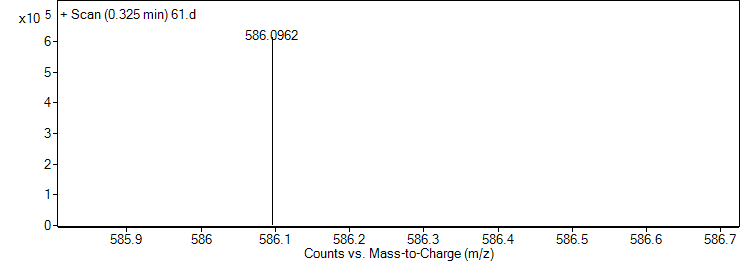
**

HRMS spectrum of the target compounds **4i**

IR spectrum of the target compounds **4j**

**
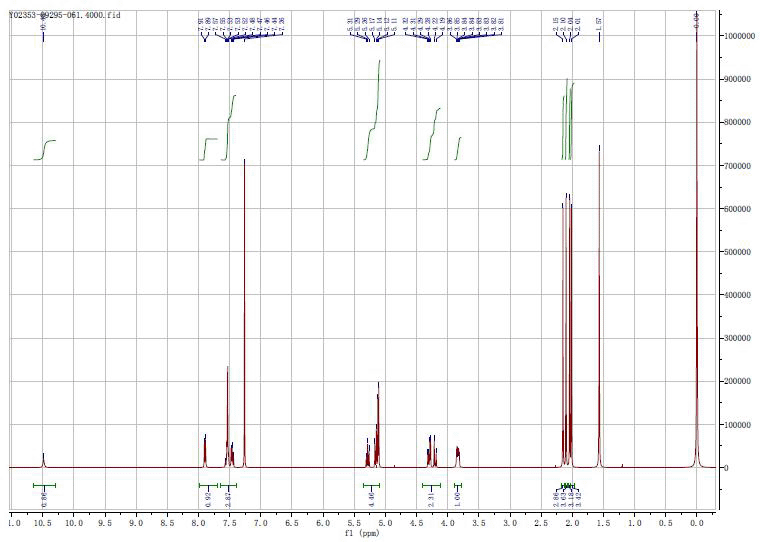
**

^1^H NMR spectrum of the target compounds **4j**

^13^C NMR spectrum of the target compounds **4j**

**
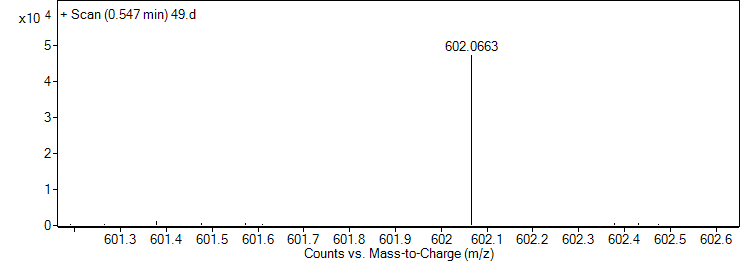
**

HRMS spectrum of the target compounds **4j**

IR spectrum of the target compounds **4k**

^1^H NMR spectrum of the target compounds **4k**

^13^C NMR spectrum of the target compounds **4k**

**
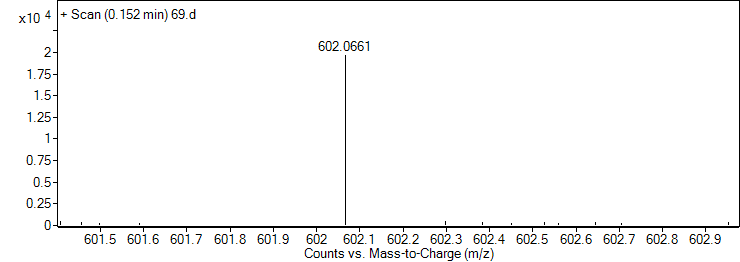
**

HRMS spectrum of the target compounds **4k**

IR spectrum of the target compounds **4l**

^1^H NMR spectrum of the target compounds **4l**

**

**

^13^C NMR spectrum of the target compounds **4l**


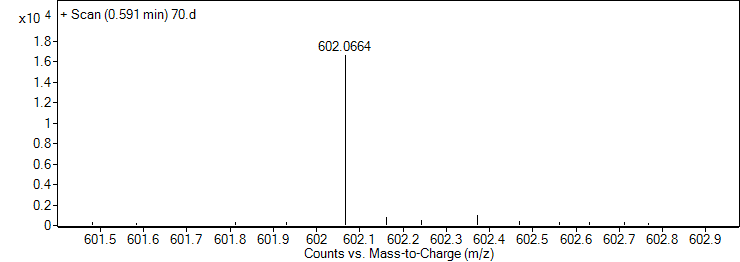
HRMS spectrum of the target compounds **4l**

IR spectrum of the target compounds **4m**

**

**

^1^H NMR spectrum of the target compounds **4m**


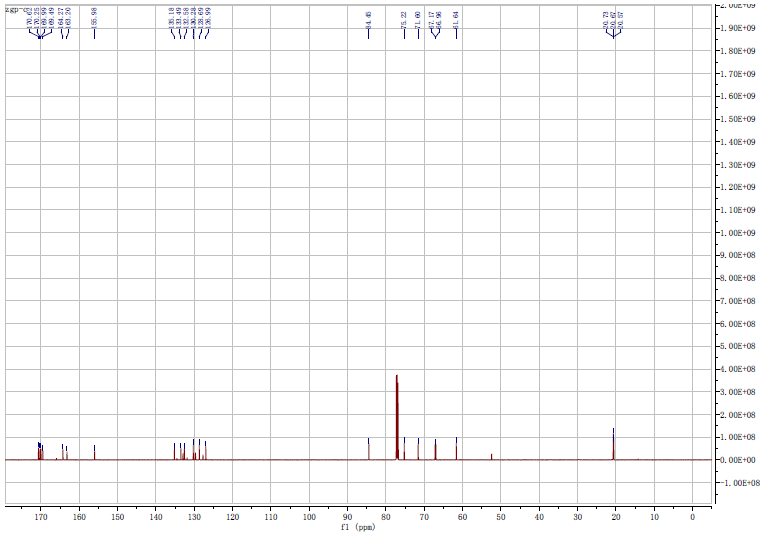


^13^C NMR spectrum of the target compounds **4m**

**
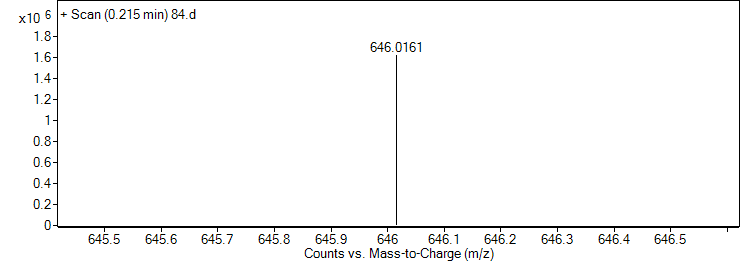
**

HRMS spectrum of the target compounds **4m**

IR spectrum of the target compounds **4n**

^1^H NMR spectrum of the target compounds **4n**

^13^C NMR spectrum of the target compounds **4n**


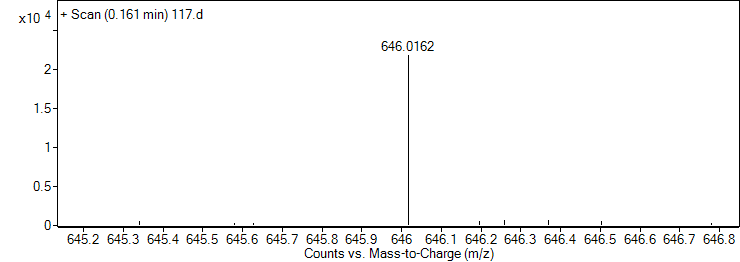
HRMS spectrum of the target compounds **4n**

IR spectrum of the target compounds **4o**

^1^H NMR spectrum of the target compounds **4o**

^13^C NMR spectrum of the target compounds **4o**

**
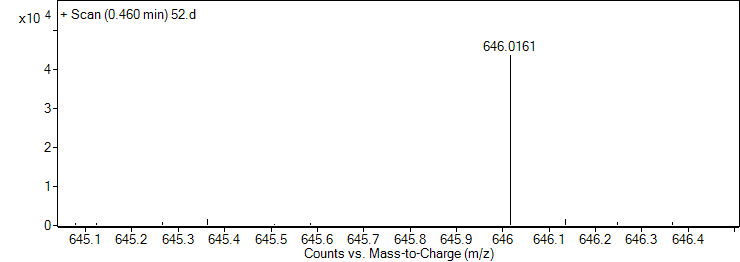
**

HRMS spectrum of the target compounds **4o**

IR spectrum of the target compounds **4p**

**
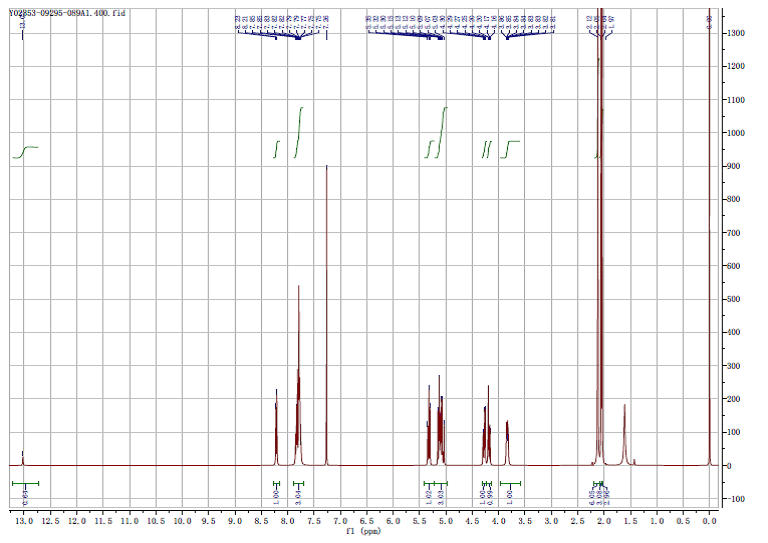
**

^1^H NMR spectrum of the target compounds **4p**

^13^C NMR spectrum of the target compounds **4p**


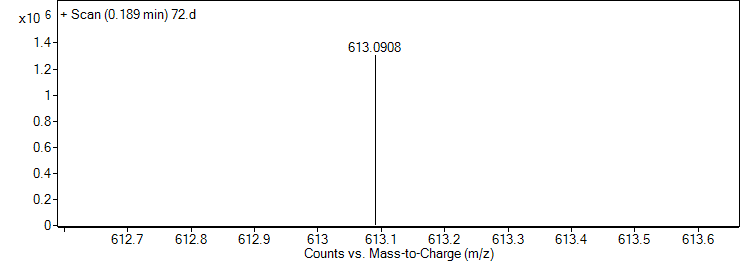
HRMS spectrum of the target compounds **4p**

IR spectrum of the target compounds **4q**

^1^H NMR spectrum of the target compounds **4q**

^13^C NMR spectrum of the target compounds **4q**

**
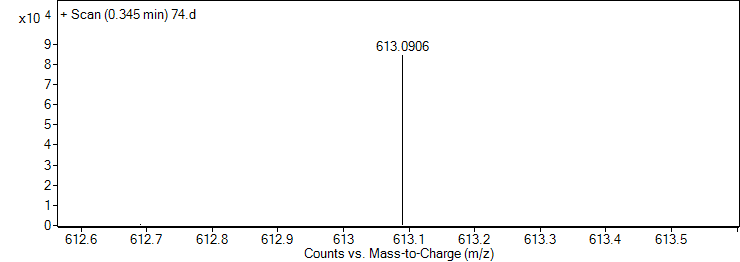
**

HRMS spectrum of the target compounds **4q**
